# Identification of Critical Phosphorylation Sites Enhancing Kinase Activity With a Bimodal Fusion Framework

**DOI:** 10.1016/j.mcpro.2024.100889

**Published:** 2024-11-30

**Authors:** Menghuan Zhang, Yizhi Zhang, Keqin Dong, Jin Lin, Xingang Cui, Yong Zhang

**Affiliations:** 1 State Key Laboratory of Cardiovascular Diseases and Medical Innovation Center, Institute for Regenerative Medicine, Department of Neurosurgery, Shanghai East Hospital, Shanghai Key Laboratory of Signaling and Disease Research, Frontier Science Center for Stem Cell Research, School of Life Sciences and Technology, Tongji University, Shanghai, China; 2 Department of Urology, School of Medicine, Xinhua Hospital Affiliated to Shanghai Jiao Tong University, Shanghai, China

**Keywords:** kinase activity, critical phosphorylation site, deep learning, phosphorylation mass spectrometry, embedding

## Abstract

Phosphorylation is an indispensable regulatory mechanism in cells, with specific sites on kinases that can significantly enhance their activity. Although several such critical phosphorylation sites (phos-sites) have been experimentally identified, many more remain to be explored. To date, no computational method exists to systematically identify these critical phos-sites on kinases. In this study, we introduce PhoSiteformer, a transformer-inspired foundational model designed to generate embeddings of phos-sites using phosphorylation mass spectrometry data. Recognizing the complementary insights offered by protein sequence data and phosphorylation mass spectrometry data, we developed a classification model, CSPred, which employs a bimodal fusion strategy. CSPred combines embeddings from PhoSiteformer with those from the protein language model ProtT5. Our approach successfully identified 77 critical phos-sites on 58 human kinases. Two of these sites, T517 on PKG1 and T735 on PRKD3, have been experimentally verified. This study presents the first systematic and computational approach to identify critical phos-sites that enhance kinase activity.

## Highlights

• Developed PhoSiteformer to capture internal relationships between phos-sites.
• Introduced CSPred, a bimodal framework combining PhoSiteformer and ProtT5 embeddings.
• Systematically identified 77 newly critical phos-sites on 58 human kinases.

## Graphical Abstract

**Figure:**
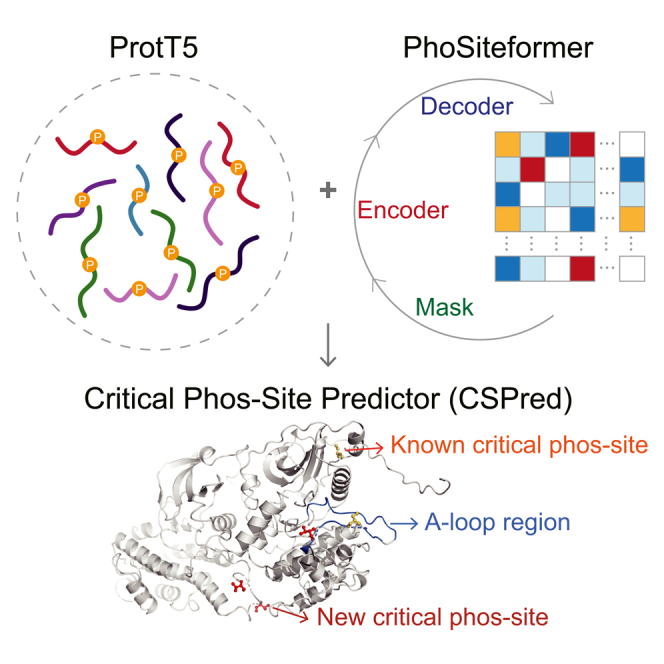

## In Brief

Phosphorylation is essential for cellular regulation, yet identifying critical phosphorylation sites (phos-sites) on kinases remains challenging. This study introduces PhoSiteformer, a model leveraging phosphorylation mass spectrometry data to learn internal relationships between phos-sites, and CSPred, a bimodal framework combining PhoSiteformer and ProtT5 embeddings to systematically identify critical sites. The approach uncovered 77 previously unreported critical phos-sites across 58 human kinases. This work advances understanding of kinase activity and regulation, providing a computational framework to uncover essential phosphorylation events.

Kinases are crucial for several key cellular processes, such as signaling, metabolism, and differentiation (1, 2). Their dysregulation is often linked to the development and progression of various diseases, especially cancer (3, 4, 5). Traditionally, disease diagnosis related to kinase dysfunction has relied on evaluating the enzyme's activity status. However, these methods confront substantial challenges due to the complexity of cellular signaling pathways and the difficulties inherent in direct enzymatic activity assays (6, 7). Decades of research have established that specific phosphorylation sites (phos-sites) are critical for kinase activity. For example, DAPK3 activation requires phosphorylation at T180, T225, and T265 (8); activation of the AMPK α subunit depends on phosphorylation at T172 (9); AurA is activated by phosphorylation at T288 on its activation loop (10); a mutation from T160 to A in CDK2 can completely abolish its kinase activity, highlighting the importance of this site (11); and the activation of P38A kinase needs the dual phosphorylation at T180 and Y182 (12). Researchers have cataloged 177 critical phos-sites across 112 human kinases in UniProt (13), which significantly enhance kinase activity. Using phospho-specific antibodies to measure such phosphorylation events has been proven to be a valuable biomarker for assessing kinase activity, providing a more direct and clinically applicable solution (6). Despite this progress, the broader application of this method is limited by the relatively small number of kinases with well-characterized and experimentally validated critical phos-sites. Considering that there are over 500 known kinases in human (14), many critical phos-sites that affect kinase activity remain unexplored. These challenges underscore the need for advanced methods capable of reliably identifying and characterizing these critical phos-sites across a broader array of kinases.

The sequences flanking critical phos-sites within kinases display unique characteristics that can be used for prediction. However, due to the high similarity among kinase sequences (14, 15), relying solely on sequence information often results in a high rate of false positives. Previously, the lack of known kinase structures was a major barrier, but advancements such as AlphaFold2 now allow for more accurate predictions of these structures (16), opening new avenues for exploring the structural patterns of critical phos-sites. Additionally, although phosphorylation mass spectrometry (phos-MS) data are continuously expanding and hold significant promise, the inherent sparsity of these data presents considerable challenges. The development of deep learning technologies, particularly Transformer models (17), offers potential solutions for effectively learning from sparse datasets. In summary, these advancements create opportunities to develop innovative computational methods that can effectively utilize the vast amounts of phos-MS data, combined with insights from protein sequence and structural information. Integrating such diverse data has the potential to significantly enhance the prediction accuracy for critical phos-sites.

Recently, significant progress has been made in protein language models based on amino acid sequences, such as the ESM series (18, 19, 20) and ProtTrans (21). These models excel at capturing secondary and tertiary structural features and exhibit excellent versatility in a wide range of downstream applications, such as revealing protein structures (20), predicting the functional impacts of sequence variants (18), and addressing reverse folding problems (22). In this study, we found that protein sequence and phos-MS data provide complementary insights into critical phos-sites. Based on this observation, we designed a classification model named CSPred (Critical Phos-Site Predictor), which concatenated embeddings derived from the protein language model ProtT5 with those generated by a deep learning model tailored for phos-MS data. Our approach identified 77 newly critical phos-sites on 58 human kinases. Two critical phos-sites including T517 on PKG1 and T735 on PRKD3 were further experimentally validated. This study represents the first systematic computational identification of critical phos-sites that can enhance kinase activity.

## Experimental Procedures

### Datasets

The “Post-translational modification” annotations for 636 reviewed human kinases were retrieved from UniProt (release 2023_01) by searching the keyword “kinase” (KW-0418) (13). Through manual screening, we collected 177 critical phos-sites that can enhance kinase activity and 55 phos-sites known to inhibit kinase activity. The sequences of human proteins, including isoforms, were also obtained from UniProt (release 2023_02) (13). Human phos-sites and kinase–substrate relationships were downloaded from PhosphoSitePlus (23). All AlphaFold Protein Data Bank coordinate files were downloaded from AlphaFold Protein Structure Database (24). Three functional regions of the kinases, including protein kinases ATP-binding region signature (PS00107), serine/threonine protein kinases active-site signature (PS00108) and tyrosine protein kinases specific active-site signature (PS00109) were downloaded from PROSITE (release 2023_03) (25). Activation loop (A-loop) annotations were derived from InterPro (26) (v.99.0), and the group information for kinases was extracted from www.kinase.com (Kincat Hsap 08.02) (27). Phos-MS data for 23 datasets across 13 cancer types were collected from CPTAC (https://pdc.cancer.gov/pdc/browse/). Accession numbers for CPTAC are listed in the supplemental Table S1.

### Calculation of Sequence and Structural Features

Sequences flanking phos-sites (±7 amino acids) were obtained. Combinations of amino acids were grouped as followed: Asx: D, N; Glx: E, Q; Xle: I, L; Positively charged: K, R, H; Negatively charged: D, E; Uncharged: N, C, Q, S, T, Y; Aromatic: F, W, Y, H; Aliphatic: V, I, L, M; Small: P, G, A, S; Hydrophilic: S, T, H, N, Q, E, D, K, R; Hydrophobic: V, I, L, F, W, Y, M; Polar: R, N, D, C, Q, E, H, K, S, T, Y; Nonpolar: A, G, I, L, M, F, P, W, V; AHR (Common residues in alpha helix motifs): A, C, Q, E, H, L, K, M; BSR (Common residues in beta sheet motifs): I, F, T, W, Y, V; RTR (Common residues in reverse turn motifs): N, D, G, P, S; and UFR (Common residues promoting unfolding or distorted regions): G, P. For k-mer identification, every sequence was scanned using a sliding window of size k, and the occurrence rate of each k-mer was calculated. Sequence motif analysis was performed using MEME (v.5.5.4) (28).

Spatial distances from phos-sites to three functional regions were defined by calculating the distances between the Cα atoms of the phos-site and the Cα atoms of conserved residue (K or D residue) using AlphaFold Protein Data Bank coordinate files. Structural features were calculated using the PSAIA program (29), including accessible surface area (ASA), protrusion index (CX) and depth index (DPX). For ASA, backbone residue attributes (sum of all backbone atom values) were obtained. For CX and DPX, total mean residue attributes (mean value of all atom values) were obtained.

### Generation of ProtT5 Embeddings

ProtT5 embeddings were generated using the pretrained protein language model, ProtT5 (ProtT5-XL-UniRef50) (21). Specifically, the sequence flanking each phos-site (±30 amino acids) was input into ProtT5 encoder, resulting in a 1024-dimensional vector representation for each residue. The overall sequence embedding was then derived by averaging all residue embeddings. The t-distributed stochastic neighbor embedding analysis of ProtT5 embeddings was performed using the Python package Scikit-learn (v.1.2.2) (30).

### Phosphorylation Enrichment Analysis

The phosphorylation enrichment analysis of substrates was performed using the Python package GSEApy (v.1.0.4) (31). We selected phos-MS data from CPTAC, including 15 datasets across 12 cancer types, each containing over 50 samples. Cancer samples from these datasets were used for the enrichment analysis. Kinase–substrate relationships from PhosphoSitePlus (23) were used as the predefined gene set. For each kinase phos-site in each dataset, samples were divided into two groups based on whether the phosphorylation level of the site was greater than 0. All phos-sites were then ranked by the fold change in the average phosphorylation level between the two groups. Normalized enrichment scores were subsequently calculated.

### Phos-MS Data Preprocessing

All datasets from CPTAC were merged to train PhoSiteformer. For duplicate samples, we retained the one with the fewest missing values, resulting in a total of 2765 samples. For all phos-sites, we first selected those located on human kinases (supplemental Fig. S2*B*). Then, based on known kinase–substrate relationships from PhosphoSitePlus and potential kinase–substrate relationships predicted by KinasePhos (32), NetworKIN (33), and PhosphoPICK (34), we selected all possible substrate phos-sites. For the remaining phos-sites, we selected those that were detected in at least 10% of the samples. This process resulted in a final set of 95,776 phos-sites. Missing values in the phos-MS data were imputed by subtracting a small number (1×10^−4^) from the minimum value observed in the data. The phos-MS data were then normalized to a range from 0 to 1 to stabilize the training process, where 0 corresponded to the original missing values.

### Construction of Machine Learning Models

Seven machine learning models were employed to predict critical phos-sites using two types of features: customized sequence and structural features, and phos-MS data. For the customized sequence and structural features, 36 features with significant differences between known critical phos-sites and non-critical phos-sites (*p*-value <0.05) were initially selected. Specifically, for 2-mers, those with a frequency greater than 0.2 in sequences flanking known critical phos-sites and a log2 fold change (log_2_FC) greater than 1.5 were chosen. Subsequently, features with correlations above 0.7 were filtered out, resulting in 28 features as model inputs. The positive dataset consisted of 177 known critical phos-sites, and the negative dataset consisted of 1770 randomly selected phos-sites from other phos-sites on the corresponding kinases.

For the phos-MS data, principal component analysis (PCA) was conducted using the Python package Scikit-learn (v.1.2.2). The first 145 principal components, which explained more than 95% of the total variance, were chosen as model inputs. The positive dataset consisted of 58 known critical phos-sites detected in the phos-MS data, and the negative dataset consisted of 580 randomly selected phos-sites from other phos-sites on kinases detected in the phos-MS data.

For both types of features, the datasets were split into training and test sets in a ratio of 8:2. The models were trained and optimized on the training set. Performance evaluation on the test set was conducted using the following metrics: the area under the receiver operating characteristic curve (AUROC), the area under the precision-recall curve (AUPRC), the F1 score, and the Matthews correlation coefficient (MCC).

### PhoSiteformer Model Architecture

PhoSiteformer is a Transformer-like model with an asymmetric encoder-decoder structure. It primarily consists of six modules: preprocessing, embedding, encoding, extending, decoding, and reconstruction.

#### Preprocessing Module

PhoSiteformer randomly masks a proportion of values in the phos-MS data, including both nonzero and zero values. Given that the proportion of zero values is much higher than that of nonzero values, PhoSiteformer masks the same number of nonzero and zero values, rather than the same proportion, to prevent the model from simply predicting all values as zero while still maintaining a low error level. After masking, positions that are either masked or have zero values are removed. The remaining unmasked positions are aligned with padding tokens to ensure a consistent maximum length within each batch.

#### Embedding Module

The embedding module processes the nonzero elements of the unmasked matrix, represented as *V*_*unmasked*_⊙(1-*M*_*zero*_). Expression embeddings and sample embeddings are projected individually. The expression embeddings, *E*, are generated using an autodiscretization strategy (35). The sample embeddings, *S*, are initialized randomly from a look-up table, *T*, similar to the positional embeddings used in natural language modeling.

$$E=AutoDis\left(V_{unmasked}⊙\left({1-M}_{zero}\right)\right)$$

S=T(samples)

#### Encoding and Extending Module

The combined expression and sample embeddings are then fed into the encoder, which is based on a traditional multihead attention Transformer. The encoder’s output embeddings, along with the mask embedding *I*_*mask*_ and zero embedding *I*_*zero*_, are subsequently passed through a fully connected (FC) layer.

$$I_{encoder-input}=E⊕S$$

$$I_{encoder-output}=Transformer\left(I_{encoder-input}\right)$$

$$I_{decoder-input}=FC\left(\left[I_{encoder-output},I_{mask},I_{zero}\right]\right)$$

where *FC* represents fully connected layer and [.] denotes concatenation.

#### Decoding and Reconstruction Module

The output embeddings are then fed into the Performer decoder, which is an optimized framework for extracting full-length features through long-sequence attention calculations. The decoder embeddings are projected to the model output *via* a FC layer to reconstruct the phos-MS data. At the masked positions, the model calculates the mean squared error loss between the predicted and the actual values.

$$I_{decoder-output}=Performer\left(I_{decoder-input}\right)$$

$$V_{reconstruct}=FC\left(I_{decoder-output}\right)$$

The preprocessed phos-MS data were split into training and validation sets with a ratio of 75:25 to train and validate PhoSiteformer.

### Generation of PhoSiteformer Embeddings

To generate PhoSiteformer embeddings, we input the phos-MS data of 5309 kinase phos-sites into the pretrained PhoSiteformer encoder (epoch 11) to obtain contextual sample embeddings. A max-pooling layer was then applied to aggregate these sample embeddings into a single embedding for each phos-site.

### Kinase–Substrate Relation Prediction

The positive dataset consisted of 3618 kinase–substrate pairs from PhosphoSitePlus. In order to obtain a more reliable negative dataset, we first generated random kinase–substrate pairs and then excluded both the known kinase–substrate pairs from PhosphoSitePlus and the potential kinase–substrate pairs predicted by KinasePhos, NetworKIN, and PhosphoPICK. We configured the ratio of positive to negative relations as either 1:1 (balanced dataset) or 1:10 (imbalanced dataset). We employed a max-pooling layer to aggregate the PhoSiteformer embeddings of the phos-sites on the kinases to derive the kinase embeddings. The embeddings of kinases and the substrate phos-sites were concatenated and fed into a multilayer perceptron (MLP). The MLP consisted of two FC linear layers, with a LayerNorm layer, ReLU activation function, and a Dropout layer in between. The final linear layer produced a single output value, termed logits. A sigmoid function was applied to convert logits into probabilities. The dataset was split into training, validation, and test sets in a ratio of 7:2:1. The model was continuously optimized until the AUROC on the validation set no longer increased. Finally, AUROC, AUPRC, F1 score, and MCC were calculated on the test set for performance evaluation.

### CSPred Model Architecture

CSPred is an integrated framework designed to identify critical phos-sites by simultaneously accepting ProtT5 embeddings and PhoSiteformer embeddings as inputs. It comprises two main modules: the feature integration module and the classifier.

#### Feature Integration Module

For phos-sites with both ProtT5 embeddings, $X_{ProtT5}^{1}$, and PhoSiteformer embeddings, *X*_*PhoSiteformer*_, CSPred concatenates these embeddings form an integrated embeddings of dimension *D*, $I_{both}\in R^{D}$. For phos-sites with only ProtT5 embeddings, $X_{ProtT5}^{2}$, CSPred employs a FC layer to expand the dimension of ProtT5 embeddings to *D* for alignment, $I_{ProtT5-only}\in R^{D}$.

$$X_{ProtT5}^{1},X_{ProtT5}^{2}\in R^{1024}\;X_{PhoSiteformer}\in R^{d}$$

$$I_{both}=\left[X_{ProtT5}^{1},X_{PhoSiteformer}\right]\in R^{D}$$

$$I_{ProtT5-only}=FC\left(X_{ProtT5}^{2}\right)\in R^{D}$$

The combined embeddings from both types, $I_{input}\in R^{D}$, are used as input embeddings for the classifier.

$$I_{input}=\left[I_{both},I_{ProtT5-only}\right]\in R^{D}$$

Here, *D*=1024+*d*, where *FC* represents fully connected layer and [.] denotes concatenation.

#### Classifier

The classifier is an MLP composed of four FC layers. Between each layer, we apply LayerNorm, ReLU activation functions, and Dropout. The first three FC layers progressively reduce the dimension by half to learn hidden representations. The final FC layer outputs logits, which are then transformed into probabilities using a sigmoid function to predict critical phos-sites. We use the BCEWithLogitsLoss function, which combines a sigmoid layer with Binary Cross Entropy Loss (BCELoss), with specific weighting to phos-sites located outside the A-loop. This loss is calculated between the predicted logits and the true labels.

$$Proba=MLP\left(I_{input}\right)$$

$$Loss=-\frac{1}{N}\sum_{i=1}^{N}\left[w_{i}\left(y_{i}\log\left(\sigma \left({\hat{y}}_{i}\right)\right)+\left(1-y_{i}\right)\log\;\left(1-\sigma \left({\hat{y}}_{i}\right)\right)\right)\right]$$

where *N* represents the number of phos-sites, ${\hat{y}}_{i}$ represents the output logits, *y*_*i*_ represents the true label (0 or 1), and *w*_*i*_ represents the weight of the phos-site. Here, *σ* is the sigmoid function, and log represents the natural logarithm.

### Identification of Critical Phos-Sites In and Out of the A-Loop

For classification models using ProtT5 embeddings and CSPred, the positive datasets for predicting critical phos-sites in and out of the A-loop included 93 and 84 known critical phos-sites, respectively. For classification models using PhoSiteformer embeddings, the positive datasets consisted of 35 and 23 known critical sites detected in the phos-MS data, respectively. The negative datasets were randomly selected from other phos-sites on kinases in the phos-MS data, maintaining a positive-to-negative sample ratio of 1:10. The datasets were then split into training, validation, and test sets with a ratio of 7:2:1. The models were optimized continuously until the AUROC on the validation sets no longer increased. Finally, AUROC, AUPRC, F1 score, and MCC were calculated on the test sets to assess performance.

### CSPred Model Training Strategy

We utilized 177 known critical phos-sites as the positive dataset, while 5251 other phos-sites on kinases from the phos-MS data were used as the negative dataset. Considering the approximate 1:10 ratio of known critical phos-sites to other phos-sites on a single kinase, we performed under-sampling of the negative dataset in each training round to maintain this ratio. Specifically, the training process comprised three rounds, and in each round, 177 positive samples remained constant, while negative samples were randomly sampled without replacement from the negative dataset. The dataset was then split into training and test sets in a ratio of 8:2, and the 5-fold cross-validation was conducted on the training set to ensure robustness. The model was continuously optimized until the AUROC on the validation set no longer increased. To balance the predictions for the number of critical phos-sites located in and out of the A-loop, we assigned different weights to out-of-A-loop phos-sites during loss calculation, ranging from 2^0^ to 2^4^. Finally, AUROC, AUPRC, F1 score, and MCC were calculated on the test set to assess performance.

For the random forest (RF) and support vector machine (SVM) displayed in supplemental Fig. S3, we adopted the same training strategy as used for CSPred. Model inputs consisted of 28 customized sequence and structural features described in the “Construction of Machine Learning Models” section, combined with the results of PCA on the phos-MS data. Here, due to the changes in the phos-MS data, we reselected the top 230 principal components, which explained more than 95% of the total variance.

### Univariate Cox Regression Analysis

We employed univariate Cox regression analysis using the Python package lifelines (36) (v.0.27.7) to identify cancer-specific critical phos-sites associated with prognosis. Due to the high number of missing values in the phos-MS data, we retained only those critical phos-sites with fewer than 50% missing values in each dataset. Missing values were then imputed from a Gaussian distribution with a mean shifted toward lower expression from the measured data distribution. Specifically, the mean of this Gaussian distribution was set as *μ*_0_ + *δσ*_0_, and the standard deviation was set as *λσ*_0_, where *μ*_0_ and *σ*_0_ represent the mean and standard deviation of the original data distribution. The shift (δ) and scale (λ) were chosen as −0.5 and 0.5, respectively, to ensure the imputed data closely aligned with the original data distribution. Subsequently, we conducted an univariate Cox regression analysis on the phosphorylation levels of critical phos-sites across 10 tumor-normal paired datasets.

### Plasmid Construction and Site-Directed Mutagenesis

The overexpression plasmid were constructed using pcDNA3.1 as the backbone, which was purchased from Shanghai Integrated Biotech Solutions Company. The target genes were amplified by PCR, where PKG1 and PRKD3 genes were connected to plasmids with FLAG tags, and HDAC5 and VASP genes were connected to plasmids with Myc tags. Site-directed mutagenesis plasmids were constructed using the QuickMutation Site-directed Mutagenesis kit (Beyotime). All plasmids were sequenced to confirm accurate gene sequences, with full plasmid sequences provided in the Supplementary Materials. The plasmids used in transfection experiment were extracted with TIANGEN Plasmid Medium Extraction Kit (TIANGEN). The concentration of plasmids was measured with a Nanodrop 2000 spectrophotometer, and the purity of plasmids was confirmed by agarose gel electrophoresis.

### Cell Lines

293T cells were obtained from Cell Bank of the Chinese Academy of Sciences and cultured in DMEM medium (Thermo Fisher) supplemented with 10% fetal bovine serum (Gibco). *Mycoplasma* contamination was tested by qPCR followed by agarose gel electrophoresis (forward primer: ACACCATGGGAGCTGGTAAT, reverse primer: CTTCTTCGACTTCCAGACCCAAGGCAT). Plasmid transfections were performed using Lipofectamine 3000 (Invitrogen). For a 6-cm cell culture dish, 10 μl Lipofectamine 3000, 10 μl p3000, and 5 μg DNA were mixed in 500 μl Opti-MEM Reduced Serum Medium (Thermo Fisher) and incubated with cells at 37 °C in an incubator with 5% CO_2_.

### Western Blot

For each group, an equal number of cells were lysed in RIPA Lysis Buffer (Thermo Fisher) 52 h after plasmid transfection. A 15 μl of each protein extract was separated by electrophoresis (130V, 70 min) on 10% Bis-Tris precast gels (EpiZyme) in MOPS buffer. The gels were then transferred to nitrocellulose membrane (300 mA, 110–130 min) and blocked with 5% skimmed milk for 1 h at room temperature. Primary antibodies were incubated overnight at 4 °C, followed by incubation with secondary antibodies for 1 h at room temperature. Unbound antibodies were washed off with PBST (1% Tween-20 v/v). All antibodies used in the experiment are listed in supplemental Table S2. The membranes were scanned using the Odyssey Sa Scanner and analyzed with Image Studio software.

### Statistical Analysis

All plots and statistical analyses were performed in Python. For unpaired samples, *p*-values were calculated using the Mann–Whitney U test. For paired samples, *p*-values were calculated using the Wilcoxon signed-rank test. Two-tailed tests were used for sequence features, while all other tests were one-tailed. When applicable, the Benjamini–Hochberg (BH) correction method was applied for multiple testing. In the figures, significance levels was indicated as follows: n.s. (not significant) for *p* > 0.05, ∗ for *p* < 0.05, ∗∗ for *p* < 0.01, ∗∗∗ for *p* < 0.001, and ∗∗∗∗ for *p* < 0.0001.

## Results

### ProtT5 Embeddings Improve Discrimination of Critical Phos-Sites

To identify sequence and structural features that distinguish critical phos-sites from non-critical phos-sites, we categorized 177 phos-sites as critical based on their role in enhancing kinase activity, while other 2737 phos-sites in the same 112 kinases were labeled as non-critical phos-sites (supplemental Table S3). We first focused on the sequence features within a range of ±7 amino acids flanking the phos-sites. Significant differences in amino acid composition between critical and non-critical phos-sites were discovered. Specifically, sequences flanking critical phos-sites showed enrichment in glycine (G), threonine (T), tyrosine (Y), cysteine (C), and tryptophan (W), while depletion for serine (S) and lysine (K) (supplemental Fig. S1*A*). Further categorization of amino acids by their physicochemical properties (37) revealed that sequence flanking critical phos-sites showed enrichments in residues typically found in β-sheet structures, as well as aromatic and hydrophobic amino acids. Conversely, sequence flanking critical phos-sites exhibited reductions in residues typically found in reverse turn motifs and were less likely to include hydrophilic or small residues (supplemental Fig. S1*B*). Employing a sliding window of length two to count all 2-mers, we identified a predominance of Y-rich and C-rich 2-mers in sequences flanking critical phos-sites, such as EY, YY, and CG (supplemental Fig. S1*C*), consistent with the observed higher Y and C content. Besides, among the 177 critical phos-sites, 39 shared a common motif (supplemental Fig. S1*D*), suggesting a specific sequence pattern associated with the criticality of the phos-sites. These results conclusively demonstrate significant differences in sequence signatures between critical and non-critical phos-sites.

**Fig. 1:**
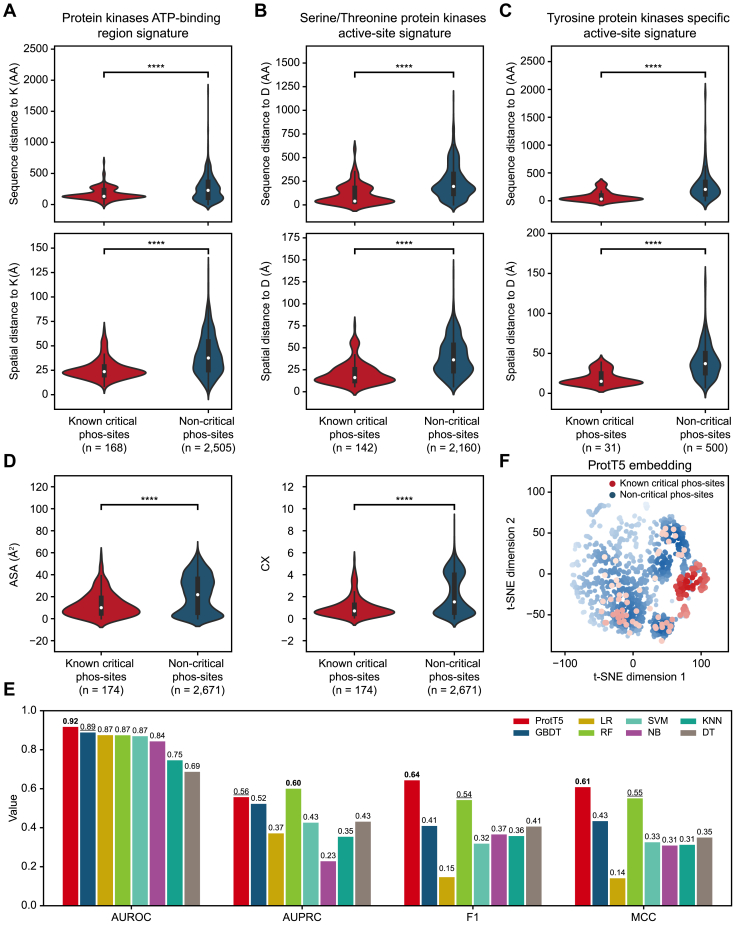
**ProtT5 embeddings improve discrimination of critical phos-sites.***A*, comparison of sequence distance (*top*) and spatial distance (*bottom*) from known critical and non-critical phosphorylation sites (phos-sites) to the conserved lysine (K) residue in the ATP-binding region signature of protein kinases. *B*, comparison of sequence distance (*top*) and spatial distance (*bottom*) from known critical and non-critical phos-sites to the conserved aspartic acid (*D*) residue in the active-site signature of serine/threonine protein kinases. *C*, comparison of sequence distance (*top*) and spatial distance (*bottom*) from known critical and non-critical phos-sites to the conserved D residue in the specific active-site signature of tyrosine protein kinases. *D*, comparison of two structural features, solvent accessible surface area (ASA) (*left*) and protrusion index (CX) (*right*), between known critical and non-critical phos-sites. Statistical difference was tested using a one-sided rank sum test. ∗∗∗∗ indicates *p* < 0.0001. *E*, performance comparison of custom sequence and structural features with ProtT5 embeddings in identifying critical phos-sites. The area under the receiver operating characteristic curve (AUROC), the area under the precision-recall curve (AUPRC), F1 score, and Matthews correlation coefficient (MCC) were calculated on the test set. *Bold* indicates the first rank, and *underline* indicates the second rank. *F*, A t-SNE representation of ProtT5 embeddings. The phos-sites are colored based on whether they are known critical phos-sites. The color intensity represents point density. DT, decision trees; GBDT, gradient boosting decision trees; KNN, K-nearest neighbors; LR, logistic regression; NB, naive bayes; RF, random forest; SVM, support vector machine; t-SNE, t-distributed stochastic neighbor embedding.

We then assessed the proximity of phos-sites in kinases to essential residues involved in ATP binding and catalysis. Specifically, we calculated the distance from phos-sites to the K residue at the end of the ATP-binding region, a key player in binding ATP (25). Critical phos-sites were significantly closer to the K residue compared to non-critical phos-sites, both in terms of the sequence (*p*-value = 1.70 × 10^−7^) and the spatial distance (*p*-value = 8.92 × 10^−26^) based on AlphaFold (24) predicted structures (Fig. 1*A*). We further calculated the distance from critical phos-sites to the conserved aspartic acid (D) residue in the catalytic domain, a crucial component for the catalytic activity of kinases (25). The patterns were consistent across kinase classes, with serine/threonine kinases showing significant proximity in sequence (*p*-value = 7.36 × 10^−23^) and spatial distance (*p*-value = 5.67 × 10^−31^) (Fig. 1*B*), and tyrosine kinases exhibiting similar trends (sequence distance *p*-value = 1.52 × 10^−10^, spatial distance *p*-value = 2.43 × 10^−11^) (Fig. 1*C*). Moreover, our analysis revealed that critical phos-sites have significantly lower solvent ASA and CX (*p*-value = 3.16×10^−10^ and 3.27×10^−14^, respectively) (Fig. 1*D*), along with a higher DPX (*p*-value = 2.48×10^−5^) (supplemental Fig. S1*E*), which is consistent with the previous report that functional phos-sites in kinases tend to be located in the interior region (38). These structural features of critical phos-sites indicate the potential roles of their proximity to essential functional residues and their interior positioning within kinases.

Taken together, we found a set of sequence and structural features that could distinguish critical phos-sites from non-critical phos-sites, suggesting the potential for the precise prediction of critical phos-sites. Subsequently, we implemented two distinct computational approaches to predict critical phos-sites (supplemental Fig. S1*F*). From our analysis, we identified 28 significant features, which were then integrated into seven different machine learning models (supplemental Table S4). Among them, the RF model demonstrated superior performance, with an AUROC of 0.87 and AUPRC of 0.60 (Fig. 1*E*). Given the robust performance of protein language models in capturing protein sequence and structural information, these models excel at encoding subtle differences. This capability allows us to explore whether their embeddings can distinguish between sequences flanking critical phos-sites and non-critical phos-sites. Here, we used the ProtT5, which is a pretrained model of ProtTrans. The t-distributed stochastic neighbor embedding visualization clearly highlighted the embedding differences between sequences flanking critical and non-critical phos-sites (Fig. 1*F*). We then input the ProtT5 embeddings into a MLP (supplemental Fig. S1*F*). This approach yielded excellent discrimination performance with an AUROC of 0.92 and an AUPRC of 0.56 (Fig. 1*E*). Therefore, ProtT5 embeddings have been proven to be more effective in distinguishing critical from non-critical phos-sites compared to custom sequence and structural features.

### PhoSiteformer: Generating Embeddings From phos-MS Data

**Fig. 2:**
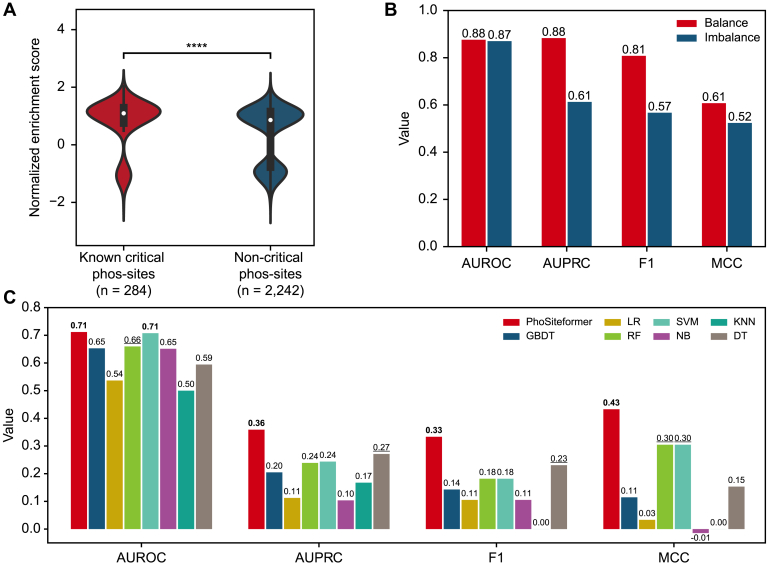
**PhoSiteformer: generating phos-site embeddings from phosphorylation mass spectrometry data.***A*, comparison of the normalized enrichment scores between known critical phos-sites and non-critical phos-sites. Statistical difference was tested using a one-sided rank sum test. ∗∗∗∗ indicates *p* < 0.0001. *B*, performance of PhoSiteformer embeddings in predicting kinase–substrate relationships on both balanced and imbalanced datasets. AUROC, AUPRC, F1 score, and MCC were calculated on the test set. *C*, performance comparison of phosphorylation mass spectrometry (phos-MS) data with PhoSiteformer embeddings in identifying critical phos-sites. AUROC, AUPRC, F1 score, and MCC were calculated on the test set. *Bold* indicates the first rank, and *underline* indicates the second rank. The seven machine learning methods used are the same as in Fig. 1F. AUROC, area under the receiver operating characteristic curve; AUPRC, area under the precision-recall curve; MCC, Matthews correlation coefficient.

We hypothesized phosphorylation at critical sites would enhance kinase activity, leading to higher phosphorylation levels of downstream substrates. To verify this, we analyzed phos-MS data from CPTAC (39) and found that samples with phosphorylation at critical sites indeed exhibited a significantly higher enrichment in substrate phosphorylation levels using gene set enrichment analysis (40) (*p*-value = 5.11 × 10^−13^) (Fig. 2*A* and supplemental Table S5).

To learn the characteristics of critical phos-sites from phos-MS data, we designed the PhoSiteformer model, inspired by the Transformer architecture (supplemental Fig. S2*A*). PhoSiteformer, featuring a scalable architecture with six million parameters, was pretrained on 95,776 phos-sites across 2765 samples (supplemental Fig. S2*B* and supplemental Table S1). It includes an embedding module and an asymmetric encoder-decoder structure. The embedding module converts continuous expression values of phos-sites into high-dimensional vectors, which are then fed into the encoder and the decoder. The encoder specifically processes embeddings from nonzero and unmasked phos-sites, while the decoder handles embeddings from all phos-sites, effectively addressing the sparsity inherent in phos-MS data. PhoSiteformer was continuously trained to reconstruct the phos-MS data, and its performance was evaluated by calculating mean square error loss, accuracy, and correlation (supplemental Fig. S2*C*), by comparing the model's predictions with the actual values at masked positions. Sample embeddings from the trained model were extracted and fed into a max-pooling layer to generate PhoSiteformer embeddings for each phos-site.

To validate whether PhoSiteformer can accurately capture the regulatory relationships between phos-sites, we utilized PhoSiteformer embeddings to predict kinase–substrate interactions. Our positive dataset included 3618 kinase–substrate pairs from PhosphoSitePlus (23). To create a robust and representative negative dataset, we removed pairs found in the positive dataset and excluded pairs with prediction scores above 0.7 from three databases: KinasePhos (32), NetworKIN (33), and PhosphoPICK (34). We then randomly assembled kinase–substrate pairs as negative dataset. The datasets used for the task were set as balanced (1:1) and unbalanced (1:10) according to the ratio of positive and negative samples. The ratio of training, validation, and test sets was set as 7:2:1. For each phos-site, we generated embedding using PhoSiteformer. For each kinase, we used a max-pooling layer to aggregate all phos-site embeddings on the kinase into one embedding. These kinase embeddings were then concatenated with the substrate phos-site embeddings to serve as inputs for the classification model. The prediction results demonstrated good performance. On the balanced dataset, the model achieved an AUROC of 0.88 and AUPRC of 0.88 (Fig. 2*B*). On the imbalanced dataset, it showed an AUROC of 0.87 and an AUPRC of 0.61 (Fig. 2*B*). Overall, these results strongly suggest that the PhoSiteformer embeddings effectively capture information about kinase–substrate relationships.

Further, we utilized PhoSiteformer embeddings to differentiate critical phos-sites from non-critical ones. By adopting an MLP for classification, the model yielded an AUROC of 0.71 and an AUPRC of 0.36 (Fig. 2*C*). For comparison, we applied PCA to reduce the dimensionality of phos-MS data from 2765 samples to 145 features. These features were then processed through seven different machine learning models (supplemental Fig. S2*D*). Notably, although the SVM model exhibited good performance with an AUROC of 0.71 and an AUPRC of 0.24 (Fig. 2*C*), its performance was slightly inferior to the results based on the PhoSiteformer embedding. Therefore, the PhoSiteformer embedding has been shown to be more effective in distinguishing critical from non-critical phos-sites.

### CSPred: Enhanced Prediction Using Combined ProtT5 and PhoSiteformer Embeddings

**Fig. 3:**
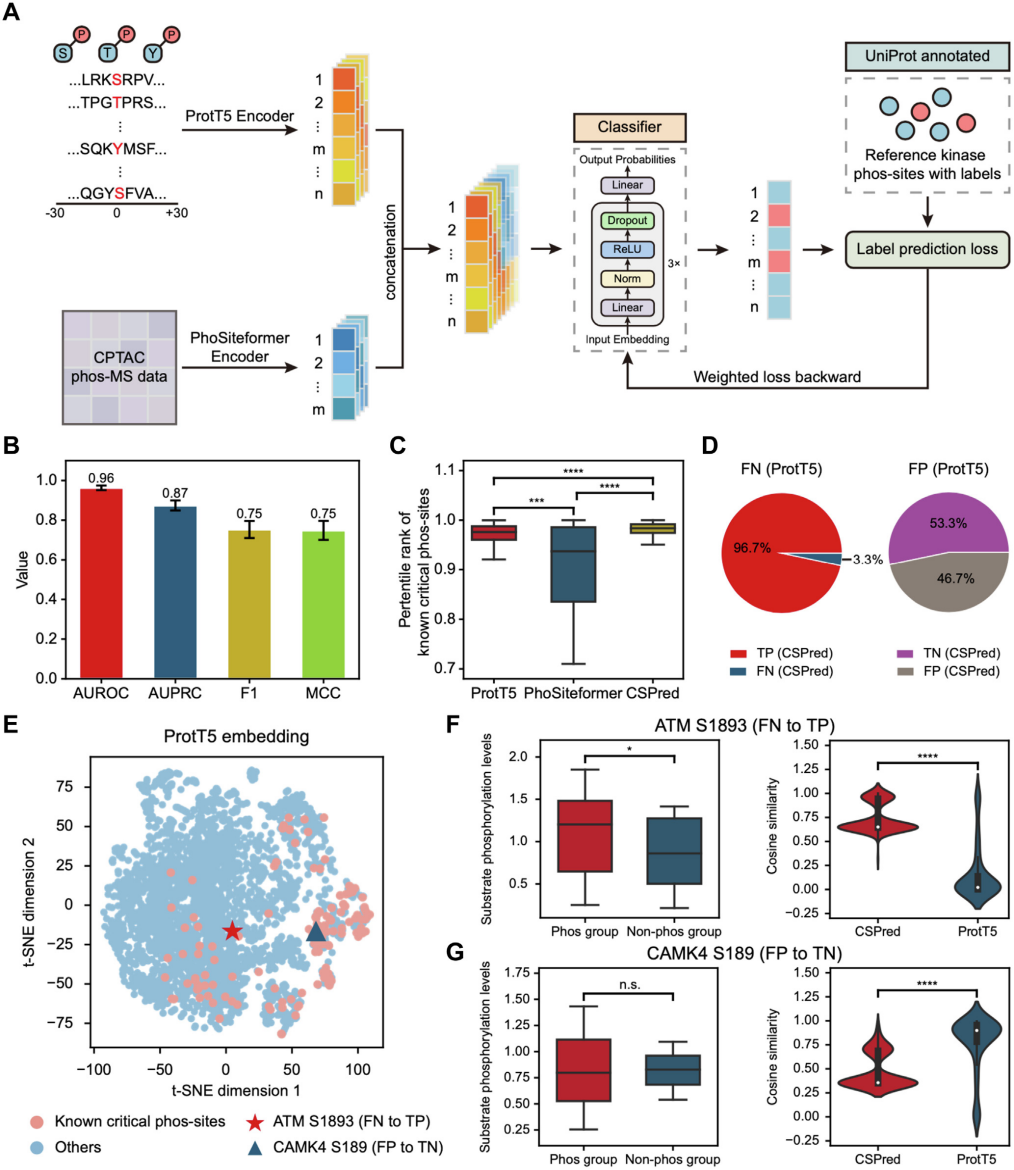
**CSPred: enhanced prediction using combined ProtT5 and PhoSiteformer embeddings.***A*, schematic illustration of CSPred. CSPred integrates two types of embeddings as input, including ProtT5 embeddings and PhoSiteformer embeddings. *B*, performance of CSPred in identifying critical phos-sites. AUROC, AUPRC, F1 score, and MCC were calculated on the test set. Data are represented as mean values across three rounds. *C*, percentile rank of known critical phos-sites as predicted by the top-performing models. *D*, CSPred predictions for instances misclassified by the model using only ProtT5 embeddings. *E*, A t-SNE visualization of ProtT5 embeddings, with two specific phos-sites, serine 1893 in ATM (ATM:S1893) and serine 189 in CAMK4 (CAMK4:S189), highlighted using deep *red* pentagram and deep *blue* triangle, respectively. ATM:S1893 is an example of an FN when using only ProtT5 embeddings but correctly classified as a TP by CSPred. CAMK4:S189 is an FP with ProtT5 embeddings alone but a TN with CSPred. *F*, *left*: comparison of downstream substrate phosphorylation levels between phosphorylated (phos-group) and unphosphorylated (non-phos group) ATM:S1893. *Right*: Comparison of cosine similarity between CSPred embeddings and ProtT5 embeddings for ATM:S1893 and other known critical phos-sites. *G*, *left*: comparison of downstream substrate phosphorylation levels between phosphorylated (phos-group) and unphosphorylated (non-phos group) CAMK4:S189. *Right*: Comparison of cosine similarity between CSPred embeddings and ProtT5 embeddings for CAMK4:S189 and known critical phos-sites. Statistical differences were determined using a one-sided rank sum test. n.s. (not significant) indicates *p* > 0.05, ∗ indicates *p* < 0.05, ∗∗∗ indicates *p* < 0.001, and ∗∗∗∗ indicates *p* < 0.0001. The results of CSPred in (*B–G*) were all based on the setting where the weight for phos-sites out of the activation loop (A-loop) was set to four for loss calculation. AUROC, area under the receiver operating characteristic curve; AUPRC, area under the precision-recall curve; FN, false negative; FP, false positive; MCC, Matthews correlation coefficient; TN, true negative; TP, true positive; t-SNE, t-distributed stochastic neighbor embedding.

Given that sequence and phos-MS data provided complementary insights into critical phos-sites, we hypothesized that integrating these two types of information could improve prediction performance. Therefore, we developed CSPred, a deep learning framework for identifying critical phos-sites that enhance kinase activity. CSPred concatenated embeddings from ProtT5 and PhoSiteformer, then fed them into a classifier built with an MLP (Fig. 3*A*). To generate ProtT5 embeddings, sequences containing ±30 amino acids flanking phos-sites were input into ProtT5, and embeddings with 1024 dimensions were extracted from the hidden states of the final encoder layer. Remarkably, all 177 known critical phos-sites could produce ProtT5 embeddings. However, only 58 of these sites were detectable in the phos-MS data and could generate corresponding PhoSiteformer embeddings, with 128 dimensions. To align the dimensions, we expanded the ProtT5-only embeddings to 1152 dimensions using a linear layer. For phos-sites with embeddings from both models, they were directly concatenated into a single 1152-dimensional vector.

The kinase A-loop can regulate kinase activity through significant conformational changes induced by phosphorylation (41). We collected A-loop annotations from InterPro (26) and observed that 52.54% of known critical phos-sites were located in the A-loop (supplemental Table S6). Given the conserved nature of the A-loop and the primary focus of ProtT5 on capturing sequence information, we divided the known critical phos-sites into two groups based on whether they are located in the A-loop. This division allowed us to compare the performance of ProtT5 and PhoSiteformer embedding in predicting these two types of critical phos-sites. The results showed that ProtT5 embedding performed well in predicting critical phos-sites both in and out of the A-loop, with particularly high accuracy for phos-sites in the A-loop, achieving an AUROC of 0.99. PhoSiteformer exhibited comparable performance for both groups. Utilizing joint embeddings, the CSPred model significantly improved prediction performance for phos-sites out of the A-loop (supplemental Fig. S3, *A* and *B*). After inputting all 177 known critical phos-sites into the CSPred, we selected the top 5% based on prediction scores and discovered that 87.16% of these sites were located in the A-loop (supplemental Fig. S3*C*), corresponding to the results when the weight was set to 2^0^. To balance the distribution of phos-sites in and out of the A-loop, we adjusted the weights of phos-sites out of the A-loop for loss calculation in the MLP training process. We tested weights ranging from 2^0^ to 2^4^ and found that a weight of 2^2^ allowed 40.57% of critical phos-sites to be out of the A-loop (supplemental Fig. S3*C*). The prediction performance remained relatively consistent across different weights (supplemental Fig. S3*D*). Therefore, we selected a final weight of 2^2^ for CSPred. This model achieved an AUROC of 0.96 ± 0.01 and an AUPRC of 0.87 ± 0.03 (Fig. 3*B*). We selected the best-performing model, with an AUROC of 0.98 and an AUPRC of 0.94. This model, which incorporates PhoSiteformer embeddings, ranked known critical phos-sites higher (Fig. 3*C*).

Given the strong results achieved using custom sequence and structural features with a RF model, as well as the promising outcomes from using phos-MS data with a SVM, we further evaluated traditional machine learning methods against deep learning approaches. Specifically, we used the 28 features from the sequence and structure data and the 230 features from the phos-MS data as inputs for RF and SVM models, respectively. CSPred consistently outperformed both RF and SVM across AUROC and AUPRC metrics (supplemental Fig. S3*E*), demonstrating that CSPred is particularly effective at predicting critical phos-sites on kinases.

Further analysis revealed that integrating PhoSiteformer embeddings allowed some false positives identified by the ProtT5-only model to be correctly reclassified as true negatives. Additionally, many false negatives were accurately reclassified as true positives (Fig. 3*D*). For example, ATM S1893 was initially labeled a false negative by using only ProtT5 embeddings due to its low cosine similarity with known critical phos-sites (Fig. 3, *E* and *F*). However, phosphorylation of ATM on S1893 significantly upregulated the phosphorylation levels of its substrate sites (Fig. 3*F*). Incorporating PhoSiteformer embeddings allowed CSPred to correctly identify this phos-site as a true positive. Conversely, CAMK4 S189 was incorrectly classified as a false positive (FP) based solely on ProtT5 embeddings because of its high cosine similarity with known critical phos-sites (Fig. 3, *E* and *G*). After integrating PhoSiteformer embeddings, CSPred correctly reclassified it as a true negative, as it does not significantly impact the phosphorylation levels of its substrate sites (Fig. 3*G*). These examples further demonstrate that CSPred, by integrating PhoSiteformer and ProtT5 embeddings, significantly enhances the predictive accuracy.

### Experimental Validation and Functional Significance of Critical Phos-Sites

**Fig. 4:**
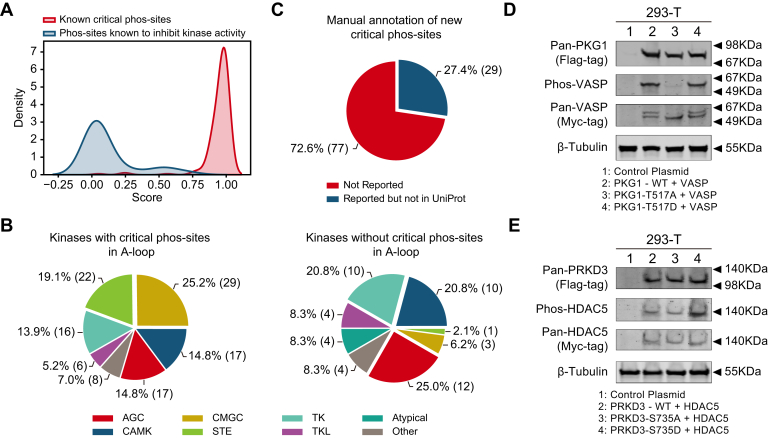
**Experimental validation and functional significance of critical phos-sites.***A*, distribution of prediction scores for known critical phos-sites and phos-sites known to inhibit kinase activity. *B*, proportion of kinase groups based on whether the kinase has critical phos-sites in the A-loop or not. *C*, proportion of new identified critical phos-sites that have been reported in the literature but not included in UniProt, compared to those not been reported. *D*, validation of threonine 517 (T517) in PKG1 by Western blotting. Aliquots of cultured 293-T cells were transfected by pcDNA3.1(+)-human VASP-3×myc, together with same amount of pcDNA3.1(+)- human PKG1-flag, pcDNA3.1(+)-human PKG1-flag-T517A, or pcDNA3.1(+)- human PKG1-flag-T517D. After 52 h, cells were lysed and assayed by Western blotting to assess pan-PKG1 level (anti-flag tag), pan-VASP level (anti-myc tag), and Ser(P)-157-VASP level. *E*, validation of serine 735 (S735) in PRKD3 *via* Western blotting. Aliquots of cultured 293-T cells were transfected by pcDNA3.1(+)-human HDAC5-3×myc, together with same amount of pcDNA3.1(+)-human PRKD3-3×flag, pcDNA3.1(+)-human PRKD3-3×flag-S735A, or pcDNA3.1(+)-human PRKD3-3×flagS735D. After 52 h, cells were lysed and assayed by Western blotting to assess pan-PRKD3 level (anti-flag tag), pan-HDAC5 level (anti-myc tag), and Ser(P)-498-HDAC5 level.

Using the CSPred model, a total of 5428 phos-sites on kinases was predicted (supplemental Table S7). The top 5%, or the 272 highest-scoring sites, were designated as high-confidence predictions. Of these, 106 are newly identified functional sites, while the remaining 166 are known critical phos-sites in UniProt (supplemental Fig. S4*A* and supplemental Table S7). To assess the reliability of our results, we collected 29 phos-sites that inhibit kinase activity in UniProt (13). The prediction scores for these phos-sites were significantly lower compared to the 177 known critical phos-sites (Fig. 4*A*). Given the crucial role of the A-loop in regulating kinase activity, we categorized kinases into two groups: those with critical phos-sites in the A-loop and those without. This categorization revealed clear differences between kinase families. The CMGC and STE families predominantly possess critical phos-sites in the A-loop, while the CAMK and TK families may lack critical phos-sites in this region (Fig. 4*B*). Previous studies have shown that certain kinases, such as EGFR-TK, DAPK-CAMK, and CAMKII, remained active even without phosphorylation of their A-loops (41, 42).

We performed univariate Cox regression analysis and identified several cancer-specific critical phos-sites associated with survival (supplemental Fig. S4*B* and supplemental Table S8). For examples, abnormal phosphorylation of MEK2:S222 and ERK3:S189 was significantly correlated with survival in patients with clear cell renal cell carcinoma and lung adenocarcinoma. Both MEK2 and ERK3 are closely related to cancer progression. Upregulation of MEK2 can activate the ERK signaling pathway, promoting clear cell renal cell carcinoma progression, and elevated levels of MEK2 in tissues are associated with lower survival rates in patients (43). ERK3 is involved in angiogenesis by regulating VEGFR2 expression, which induces endothelial cell migration, proliferation, and vascular formation (44).

Through careful manual curation of the literature, we found that 29 of the 106 newly identified critical phos-sites have been experimentally validated in previous studies but have not yet been recorded in UniProt (13) (Fig. 4*C* and supplemental Table S9). Among the remaining 77 sites, we selected those for experimental verification that met specific criteria: the kinase having only one critical phos-site, and the availability of antibodies for the corresponding substrate phos-site. Western blot analysis provided further evidence. Both the wildtype kinase PKG1 and its phosphorylation-mimicking mutant PKG1 T517D led to the phosphorylation of the substrate VASP at S157, whereas the nonphosphorylatable mutant PKG1 T517A hardly caused any phosphorylation of VASP S157, indicating that the phos-site PKG1 T517 is essential for PKG1 kinase activity (Fig. 4*D* and supplemental Fig. S4, *C* and *D*). Notably, the observed phosphorylation of VASP S157 by the wildtype PKG1 may result from endogenous phosphorylation of PKG1 at T517 upon plasmid transfection into 293-T cells. The wildtype kinase PRKD3 promoted the phosphorylation of the substrate HDAC5 at S498. The phosphorylation-mimicking mutant T735D further increased the phosphorylation levels of HDAC5 S498, while the nonphosphorylatable mutant T735A significantly reduced it (Fig. 4*E* and supplemental Fig. S4, *E* and *F*). These findings from multiple perspectives convincingly validate the reliability of the predicted critical phos-sites.

## Discussion

Phos-MS technology provides unparalleled opportunities for the large-scale identification and quantification of protein phosphorylation events. In a single experiment, thousands of phos-sites can be identified. The phosphorylation status observed at each site in phos-MS data reflects the enzymatic activity of upstream kinases, allowing kinase activity to be inferred from the phosphorylation levels of their substrates. Estimating kinase activity primarily relies on the Kolmogorov–Smirnov statistical principle, which uses phos-MS data to determine statistical significance by comparing the distribution differences between phos-sites associated with a specific kinase and the rest of the identified phospho-sites. Kinase–substrate enrichment analysis leverages this principle and employs Z-tests to assess statistical significance (45). Additionally, tools commonly used for pathway enrichment analysis, such as gene set enrichment analysis (40), can also be used to estimate kinase activity. Therefore, phos-MS data provide valuable insights into kinase–substrate relationships. With the continuous accumulation of the data and advancements in deep learning technology, we can learn embeddings of phos-sites from phos-MS data. In this study, we developed a transformer-inspired model named PhoSiteformer, which offers a new perspective for identifying and studying the functions of phos-sites. We confirmed that PhoSiteformer embeddings effectively capture information about kinase–substrate relationships.

Additionally, protein language models trained on extensive sequence data, such as the ESM series (18, 19, 20) and ProtTrans (21), provide powerful tools for gaining deeper insights into protein features. We have adopted an innovative dual-modal fusion strategy that combines PhoSiteformer embeddings with ProtT5 embeddings. This approach effectively leverages the unique insights provided by phos-MS data along with the advantages of protein sequence and structural information. When CSPred is fed with dual-modal embeddings, it can identify critical phos-sites that significantly enhance kinase activity, outperforming single-modal embeddings.

Our research encounters three main limitations. First, we relied on a phos-MS dataset consisting of 2765 samples, encompassing 5309 potential phos-sites for 350 kinases. However, due to the transient nature of phosphorylation events and the sparsity of phos-MS data, PhoSiteformer might miss capturing critical phos-sites or the activity of certain kinases under specific conditions. This partly explains why we could only predict 77 critical sites on 58 human kinases, emphasizing our constraints in fully comprehending the kinase phosphorylation landscape. Second, considering the diverse structures of kinases in their inactive states and their similarities in active states (1), we attempted to validate newly identified critical phos-sites through structural prediction. However, a longstanding challenge in protein prediction is the difficulty in inferring overall conformational changes from a single phos-site. This strategy was not effectively implemented in our study. Last, although these newly identified critical phos-sites exhibit several characteristic dimensions with known critical phos-sites, their functional significance still requires further validation through large-scale experiments.

Although PhoSiteformer currently utilizes a phos-MS dataset comprising only 2765 samples, it is designed with future data growth in mind. PhoSiteformer adopts a transformer-like architecture, featuring an asymmetric encoder-decoder structure with the performer as part of the decoder component. This design aims to enhance computational efficiency when handling large-scale data, strengthen pretraining capability for more extensive datasets, and improve both accuracy and efficiency in analyzing sparse phos-MS data. This forward-thinking architectural design ensures that PhoSiteformer remains flexible and scalable, capable of accommodating future research needs as data volumes expand.

## Data availability

All datasets analyzed in this study are publicly available (see the Experimental Procedures for details). The source codes of PhoSiteformer and CSPred are publicly available at https://github.com/TongjiZhanglab/CSPred.

## Supplemental data

This article contains supplemental data.

## Conflict of interest

The authors declare no competing interests.
