# Supplementary material for: Identification of Critical Phosphorylation Sites Enhancing Kinase Activity With a Bimodal Fusion Framework
Source: Mol Cell Proteomics. 2024 Nov 30;24(1):100889. doi: 10.1016/j.mcpro.2024.100889 (PMC11774822; doi:10.1016/j.mcpro.2024.100889)
Supplement: Supplemental Figs. S1–S4 [file mmc1.pdf]

Supplementary Figures and Figure legends

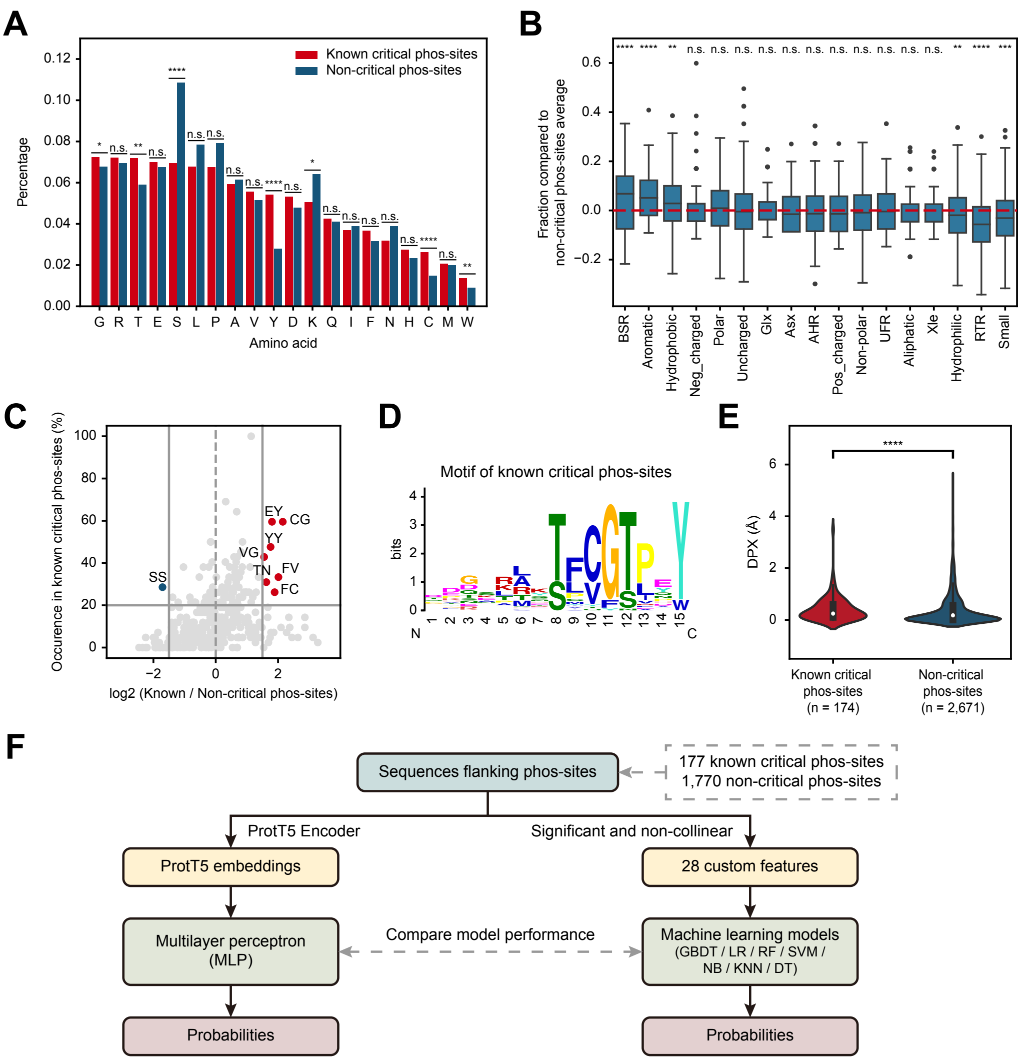

Figure S1. Sequence and Structural Features of Known Critical Phos-sites (related to Figure 1). (A) Average fraction of each amino acid in sequences flanking known critical and non-critical phosphorylation sites (phos-sites) (±7 amino acids). Statistical differences were tested using a two-sided rank sum test. A: Alanine, C: Cysteine, D: Aspartic Acid, E: Glutamic Acid, F: Phenylalanine, G: Glycine, H: Histidine, I: Isoleucine, K: Lysine, L: Leucine, M: Methionine, N: Asparagine, P: Proline, Q: Glutamine, R: Arginine, S: Serine, T: Threonine, V: Valine, W: Tryptophan, Y: Tyrosine. **(B)** Comparison of physicochemical feature fractions in sequences flanking known critical phos-sites versus non-critical phos-sites (±7 amino acids). The red dashed line at 0.0 indicates the average physicochemical feature fractions for sequences flanking non-critical phos-sites. The physicochemical features are grouped combinations of amino acids, categorized as: Asx: D, N, Glx: E, Q, Xle: I, L, Positively charged (Pos_chrged): K, R, H, Negatively charged (Neg_charged): D, E, Uncharged: N, C, Q, S, T, Y, Aromatic: F, W, Y, H, Aliphatic: V, I, L, M, Small: P, G, A, S, Hydrophilic: S, T, H, N, Q, E, D, K, R, Hydrophobic: V, I, L, F, W, Y, M, Polar: R, N, D, C, Q, E, H, K,

S, T, Y, Non-polar: A, G, I, L, M, F, P, W, V, AHR: A, C, Q, E, H, L, K, M, BSR: I, F, T, W, Y, V, RTR: N, D, G, P, S, UFR: G, P. Statistical differences were tested using a two-sided rank sum test. **(C)** Comparison of the occurrence of 2-mers in sequences flanking known critical and non-critical phos-sites (±7 amino acids). A sliding window of two amino acids was used to count the number of each 2-mer. The occurrence of each 2-mer was quantified as the ratio of its count to the total number of 2-mers across all sequences flanking known critical or non-critical phos-sites. The log2 fold change in the occurrence of each 2-mer between sequences flanking known critical and non-critical phos-sites was calculated to highlight differences in occurrence. **(D)** A consensus motif identified from sequences flanking known critical phos-sites (±7 amino acids) using MEME. **(E)** Comparison of the depth index (DPX) between known critical and non-critical phos-sites. Statistical differences were tested using a one-sided rank sum test, with significance levels indicated as follows: n.s. (not significant) for p-value > 0.05, * for $p < 0.05$, ** for $p < 0.01$, *** for $p < 0.001$, and **** for $p < 0.0001$. **(F)** Flowchart for comparing the performance of custom sequence and structural features with ProtT5 embeddings in identifying critical phos-sites. The comparison results are presented in Figure 1E.

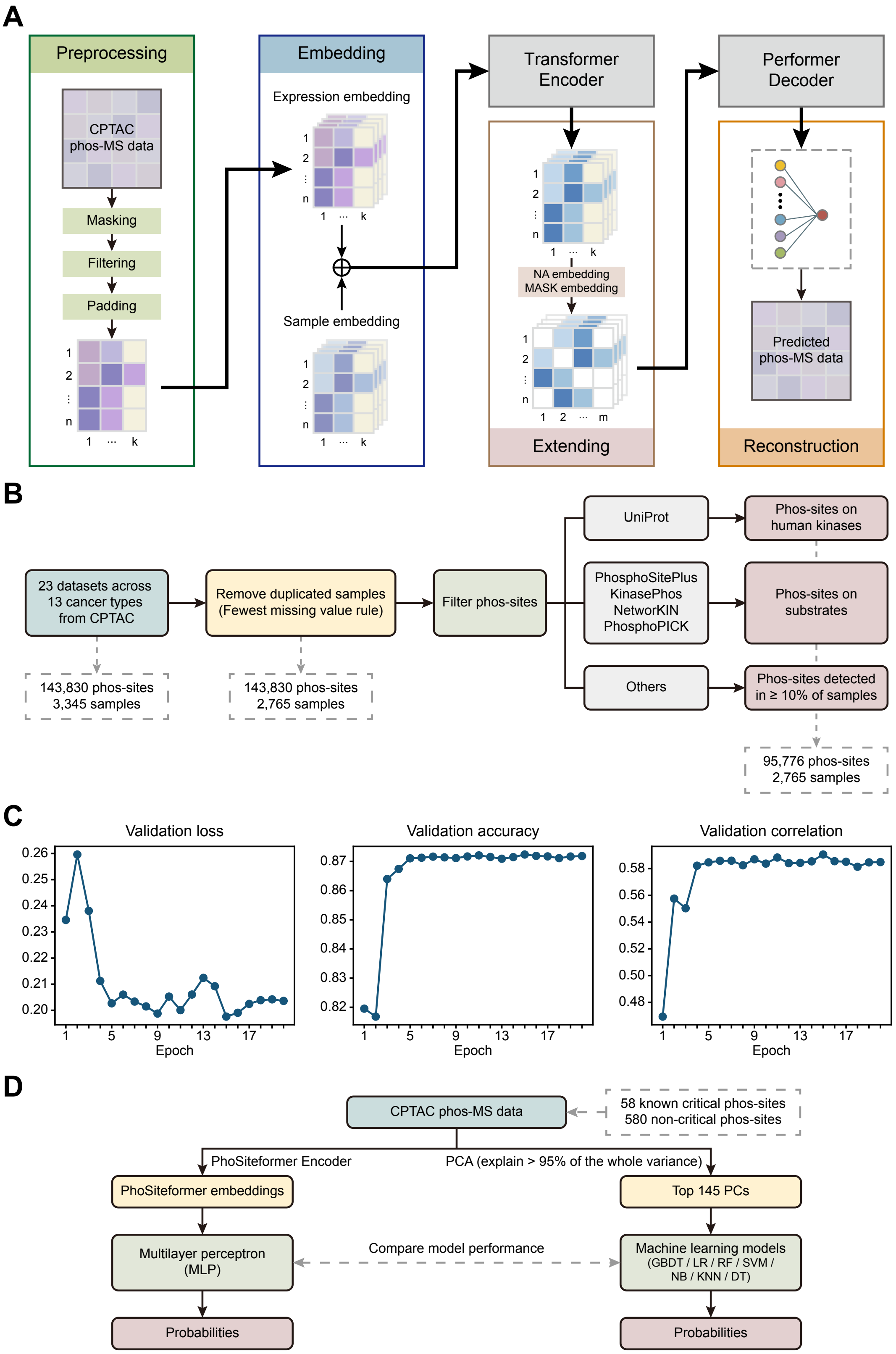

Figure S2. PhoSiteformer Model Architecture and Training Process (related to Figure 2). (A) Schematic view of PhoSiteformer. PhoSiteformer primarily consists of preprocessing, embedding, encoding, extending, decoding, and reconstruction modules. In the preprocessing stage, a portion of values in the phos-MS data was randomly

masked, including both zero and non-zero values. Subsequently, the masked and zero values were filtered, and padding tokens were added to ensure the consistent maximum length within each batch. In the embedding stage, the expression value and sample embeddings were projected separately. Then, the expression and sample embeddings were element-wise added to form the input embeddings for encoder. In the extending stage, the encoder embeddings were combined with mask embedding and zero embedding. The combined embeddings were then fed into decoder. In the reconstruction stage, the decoder embeddings were processed through a linear layer to output the predicted expression values. $\oplus$ represents element-wise addition. **(B)** Flowchart for Phos-MS data preprocessing. The preprocessed phos-MS data was split into training and validation sets with a ratio of 75:25 to train and validate PhoSiteformer. **(C)** Performance evaluation of PhoSiteformer during a 20-epoch training stage. Mean square error (MSE) loss (left), accuracy (middle) and correlation (right) were calculated for masked positions. Results are displayed for the validation set only. **(D)** Flowchart for comparing the performance of phos-MS data with PhoSiteformer embeddings in identifying critical phos-sites. The comparison results are presented in Figure 2C.

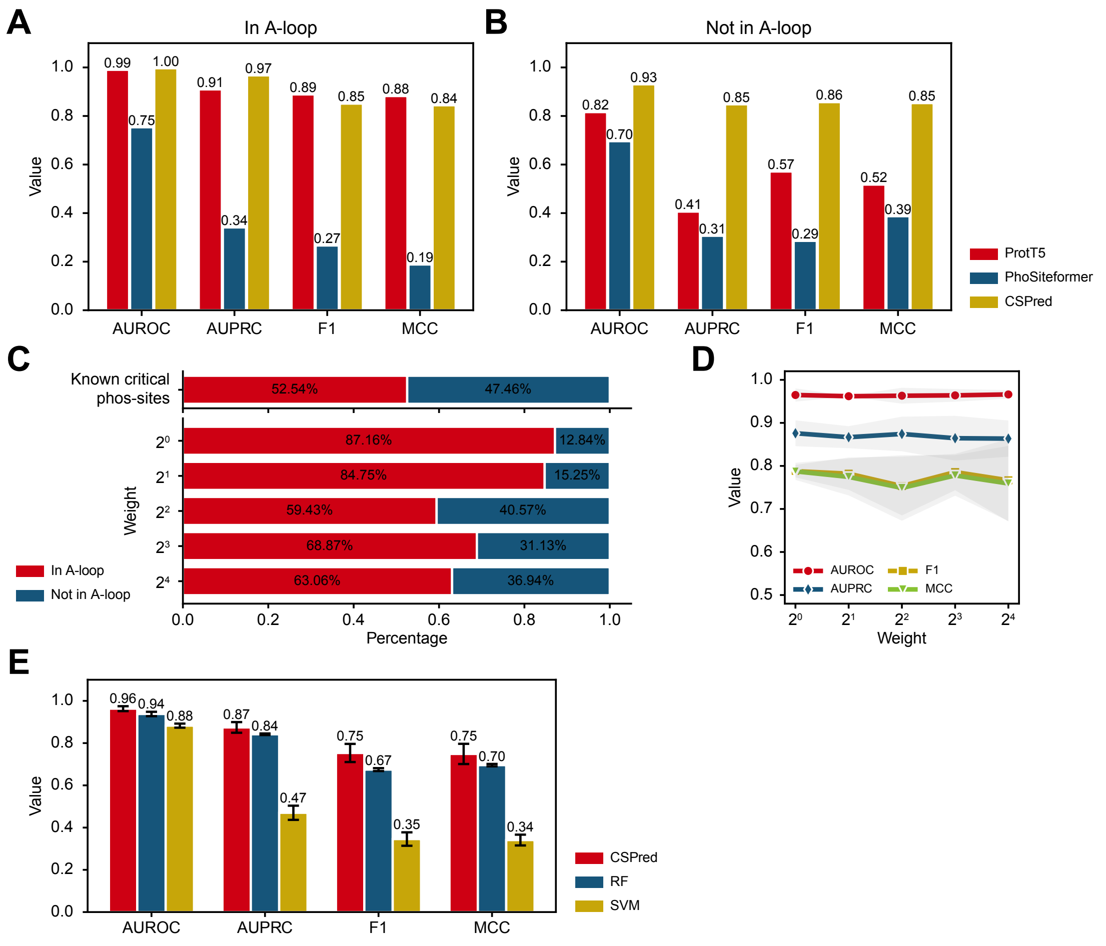

Figure S3. Performance Evaluation of CSPred in Identifying Critical Phos-sites In and Out of the A-loop (related to Figure 3). (A) Comparison of performance using ProtT5 embeddings, PhoSiteformer embeddings, and their combination (as implemented by CSPred) for identifying critical phos-sites in the activation loop (A-loop). The area under the receiver operating characteristic curve (AUROC), the area under the precision-recall curve (AUPRC), F1 score and Matthews correlation coefficient (MCC) were calculated on the test set. **(B)** Comparison of performance using ProtT5 embeddings, PhoSiteformer embeddings, and their combination (as implemented by CSPred) for identifying critical phos-sites out of the A-loop. AUROC, AUPRC, F1 score and MCC were calculated on the test set. **(C)** Proportion of known (upper panel) and new identified (lower panel) critical phos-sites in and out of the A-loop. New critical phos-sites were identified as those with predicted scores in the top 5%, ranked from high to low. "Weight" indicates the value assigned to phos-sites out of the A-loop for loss calculation. **(D)** Performance of CSPred with different weights. AUROC, AUPRC, F1 score and MCC were calculated on the test set. Lines represent mean values across three rounds, and gray shadows represent 95% confidence intervals for mean values. Weight settings are consistent with those used in (C). **(E)** Performance comparison of CSPred with traditional machine learning models. The random forest (RF) and support vector machine (SVM) models were trained

by combining 28 significant and non-collinear custom sequence and structural features with the first 230 principal components (PCs) of the phos-MS data, which explains > 95% of the total variance.

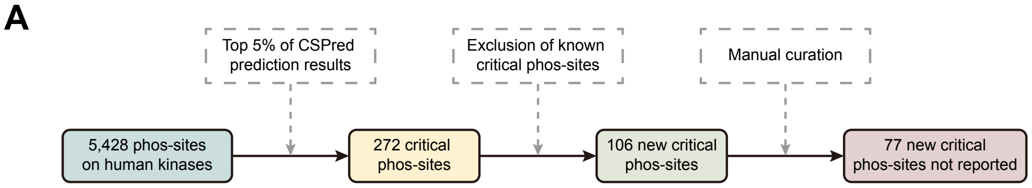

B

Dataset	Critical phos-site	Hazard ratio	95% CI	P-value	BH-adjusted p-value
CCRCC	MEK2:S222	4.65	1.86-11.63	1.03E-03	0.02
	ERK3:S189	2.25	1.36-3.73	1.70E-03	0.02
HC	PKM:S37	1.70	1.24-2.31	8.61E-04	0.02
	CDK9:S347	1.42	1.03-1.97	3.16E-02	0.19
	PRP4:Y849	1.72	1.10-2.68	1.63E-02	0.13
	ERK3:S189	1.64	1.15-2.35	6.28E-03	0.08
LA	PRKD3:S252	0.30	0.11-0.80	1.67E-02	0.12
	PKM:S37	4.30	1.54-11.96	5.27E-03	0.11
	MEK2:S222	2.51	1.23-5.11	1.13E-02	0.11
	MEK1:S218	2.51	1.23-5.11	1.13E-02	0.11
	ERK3:S189	1.90	1.02-3.54	4.24E-02	0.20
	NOL9:S487	0.33	0.13-0.87	2.45E-02	0.14
PDA	CAMK2B:T287	0.79	0.66-0.94	7.35E-03	0.17

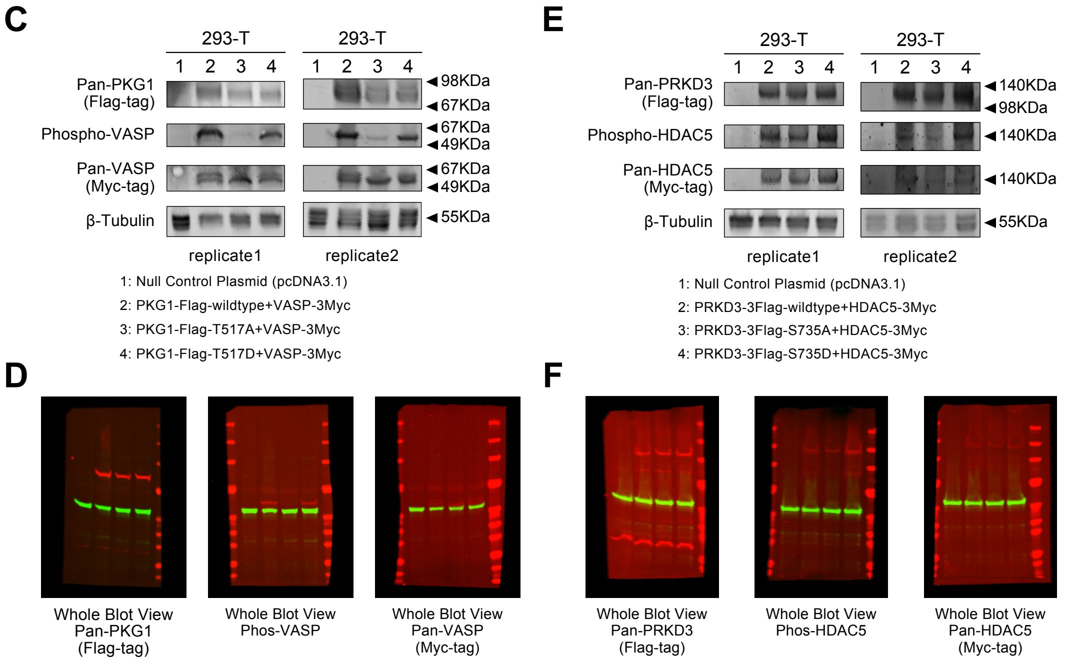

Figure S4. Clinical implications of critical phos-sites and experimental validation of two new identified critical phos-sites (related to Figure 4). (A) Flowchart for the prediction and screening of new critical phos-sites. **(B)** Results of univariate Cox regression analysis for critical phos-sites, showing only those with Benjamini-Hochberg (BH)-adjusted p-values < 0.2. CI: Confidence Interval, CCRCC: Clear Cell Renal Cell Carcinoma, HC: Hepatocellular Carcinoma, LA: Lung Adenocarcinoma,

PDA: Pancreatic Ductal Adenocarcinoma. **(C)** Two independent western blot experiments for threonine 517 (T517) in PKG1, corresponding to Figure 4D. **(D)** Original western blot images for bands shown in Figure 4C. **(E)** Two independent western blot experiments for serine 735 (S735) in PRKD3, corresponding to Figure 4F. **(F)** Original western blot images for bands shown in Figure 4E.
