# Supplementary material for: Identification of Critical Phosphorylation Sites Enhancing Kinase Activity With a Bimodal Fusion Framework
Source: Mol Cell Proteomics. 2024 Nov 30;24(1):100889. doi: 10.1016/j.mcpro.2024.100889 (PMC11774822; doi:10.1016/j.mcpro.2024.100889)
Supplement: Supplemental Data 1 [file mmc3.pdf]

```
LOCUS       Exported                9392 bp ds-DNA    circular SYN
22-1月-2024
DEFINITION  .
ACCESSION   .
VERSION     .
KEYWORDS    Untitled 100
SOURCE      synthetic DNA construct
  ORGANISM  synthetic DNA construct
REFERENCE   1  (bases 1 to 9392)
  AUTHORS   111111111
  TITLE     Direct Submission
  JOURNAL   Exported 2024年1月22日 from SnapGene 2.3.2
            http://www.snapgene.com
FEATURES             Location/Qualifiers
     source          1..9392
                     /organism="synthetic DNA construct"
                     /mol_type="other DNA"
     enhancer        50..429
                     /note="CMV enhancer"
                     /note="human cytomegalovirus immediate early
enhancer"
     promoter        430..633
                     /note="CMV promoter"
                     /note="human cytomegalovirus (CMV) immediate
early
                     promoter"
     CDS             4055..4144
                     /codon_start=1
                     /product="3 tandem Myc epitope tags"
                     /note="3xMyc"
                     /translation="EQKLISEEDLEQKLISEEDLEQKLISEEDL"
     misc_feature    4168..4756
                     /note="WPRE"
                     /note="woodchuck hepatitis virus
posttranscriptional
                     regulatory element"
     CDS             complement(4639..4650)
                     /codon_start=1
                     /product="Factor Xa recognition and cleavage
site"
                     /note="Factor Xa site"
                     /translation="IEGR"
     polyA_signal    4822..4870
                     /note="HSV TK poly(A) signal"
                     /note="herpesvirus thymidine kinase
polyadenylation signal"
     rep_origin      5072..5500
                     /direction=RIGHT
                     /note="f1 ori"
                     /note="f1 bacteriophage origin of replication;
arrow
                     indicates direction of (+) strand synthesis"
     promoter        5514..5843
                     /note="SV40 promoter"
```

```
                     /note="SV40 enhancer and early promoter"
     rep_origin      5694..5829
                     /note="SV40 ori"
                     /note="SV40 origin of replication"
     CDS             5910..6704
                     /codon_start=1
                     /gene="aph(3')-II (or nptII)"
                     /product="aminoglycoside phosphotransferase
from Tn5"
                     /note="NeoR/KanR"
                     /note="confers resistance to neomycin,
kanamycin, and G418
                     (Geneticin(R))"
                     /
translation="MIEQDGLHAGSPAAWVERLFGYDWAQQTIGCSDAAVFRLSAQGRP

VLFVKTDLSGALNELQDEAARLSWLATTGVPCAAVLDVVTEAGRDWLLLGEVPGQDLLS

SHLAPAEKVSIMADAMRRLHTLDPATCPFDHQAKHRIERARTRMEAGLVDQDDLDEEHQ

GLAPAELFARLKARMPDGEDLVVTHGDACLPNIMVENGRFSGFIDCGRLGVADRYQDIA
                     LATRDIAEELGGEWADRFLVLYGIAAPDSQRIAFYRLLDEFF"
     polyA_signal    6880..7001
                     /note="SV40 poly(A) signal"
                     /note="SV40 polyadenylation signal"
     primer_bind     complement(7050..7066)
                     /note="M13 rev"
                     /note="common sequencing primer, one of
multiple similar
                     variants"
     protein_bind    7074..7090
                     /bound_moiety="lac repressor encoded by lacI"
                     /note="lac operator"
                     /note="The lac repressor binds to the lac
operator to
                     inhibit transcription in E. coli. This
inhibition can be
                     relieved by adding lactose or
                     isopropyl-beta-D-thiogalactopyranoside (IPTG)."
     promoter        complement(7098..7128)
                     /note="lac promoter"
                     /note="promoter for the E. coli lac operon"
     protein_bind    7143..7164
                     /bound_moiety="E. coli catabolite activator
protein"
                     /note="CAP binding site"
                     /note="CAP binding activates transcription in
the presence
                     of cAMP."
     rep_origin      complement(7452..8040)
                     /direction=LEFT
                     /note="ori"
                     /note="high-copy-number ColE1/pMB1/pBR322/pUC
origin of
```

```
                     replication"
     CDS             complement(8211..9071)
                     /codon_start=1
                     /gene="bla"
                     /product="beta-lactamase"
                     /note="AmpR"
                     /note="confers resistance to ampicillin, 
carbenicillin, and
                     related antibiotics"
                     /
translation="MSIQHFRVALIPFFAAFCLPVFAHPETLVKVKDAEDQLGARVGYI

ELDLNSGKILESFRPEERFPMMSTFKVLLCGAVLSRIDAGQEQLGRRIHYSQNDLVEYS

PVTEKHLTDGMTVRELCSAAITMSDNTAANLLLTTIGGPKELTAFLHNMGDHVTRLDRW

EPELNEAIPNDERDTTMPVAMATTLRKLLTGELLTLASRQQLIDWMEADKVAGPLLRSA

LPAGWFIADKSGAGERGSRGIIAALGPDGKPSRIVVIYTTGSQATMDERNRQIAEIGAS
                     LIKHW"
     promoter        complement(9072..9176)
                     /gene="bla"
                     /note="AmpR promoter"
ORIGIN
        1 gttaggcgtt ttgcgctgct tcgcgatgta cgggccagat atacgcgttg 
acattgatta
       61 ttgactagtt attaatagta atcaattacg gggtcattag ttcatagccc 
atatatggag
      121 ttccgcgtta cataacttac ggtaaatggc ccgcctggct gaccgcccaa 
cgacccccgc
      181 ccattgacgt caataatgac gtatgttccc atagtaacgc caatagggac 
tttccattga
      241 cgtcaatggg tggagtattt acggtaaact gcccacttgg cagtacatca 
agtgtatcat
      301 atgccaagta cgccccctat tgacgtcaat gacggtaaat ggcccgcctg 
gcattatgcc
      361 cagtacatga ccttatggga ctttcctact tggcagtaca tctacgtatt 
agtcatcgct
      421 attaccatgg tgatgcggtt ttggcagtac atcaatgggc gtggatagcg 
gtttgactca
      481 cggggatttc caagtctcca ccccattgac gtcaatggga gtttgttttg 
gcaccaaaat
      541 caacgggact ttccaaaatg tcgtaacaac tccgccccat tgacgcaaat 
gggcggtagg
      601 cgtgtacggt gggaggtcta tataagcaga gctctggcta gcgtttaaac 
ttaagcttgg
      661 taccgagctc ggatccgcca ccatgaactc tcccaacgag tcggatggga 
tgtcaggtcg
      721 ggaaccatcc ttggaaatcc tgccgcggac ttctctgcac agcatccctg 
tgacagtgga
      781 ggtgaagccg gtgctgccaa gagccatgcc cagttccatg gggggtgggg 
gtggaggcag
      841 ccccagccct gtggagctac ggggggctct ggtgggctct gtggacccca 
cactgcggga
```

901 gcagcaactg cagcaggagc tcctggcgct caagcagcag cagcagctgc agaagcagct
961 cctgttcgct gagttccaga aacagcatga ccacctgaca aggcagcatg aggtccagct
1021 gcagaagcac ctcaagcagc agcaggagat gctggcagcc aagcagcagc aggagatgct
1081 ggcagccaag cggcagcagg agctggagca gcagcggcag cgggagcagc agcggcagga
1141 agagctggag aagcagcggc tggagcagca gctgctcatc ctgcggaaca aggagaagag
1201 caaagagagt gccattgcca gcactgaggt aaagctgagg ctccaggaat tcctcttgtc
1261 gaagtcaaag gagcccacac caggcggcct caaccattcc ctcccacagc accccaaatg
1321 ctggggagcc caccatgctt ctttggacca gagttcccct ccccagagcg gcccccctgg
1381 gacgcctccc tcctacaaac tgcctttgcc tgggccctac gacagtcgag acgacttccc
1441 cctccgcaaa acagcctctg aacccaactt gaaagtgcgt tcaaggctaa aacagaaggt
1501 ggctgagcgg agaagcagtc ccctcctgcg tcgcaaggat gggactgtta ttagcacctt
1561 taagaagaga gctgttgaga tcacaggtgc cgggcctggg gcgtcgtccg tgtgtaacag
1621 cgcacccggc tccggcccca gctctcccaa cagctcccac agcaccatcg ctgagaatgg
1681 ctttactggc tcagtcccca acatccccac tgagatgctc cctcagcacc gagccctccc
1741 tctggacagc tcccccaacc agttcagcct ctacacgtct ccttctctgc ccaacatctc
1801 cctagggctg caggccacgg tcactgtcac caactcacac ctcactgcct ccccgaagct
1861 gtcgacacag caggaggccg agaggcaggc cctccagtcc ctgcggcagg gtggcacgct
1921 gaccggcaag ttcatgagca catcctctat tcctggctgc ctgctgggcg tggcactgga
1981 gggcgacggg agcccccacg ggcatgcctc cctgctgcag catgtgctgt tgctggagca
2041 ggcccggcag cagagcaccc tcattgctgt gccactccac gggcagtccc cactagtgac
2101 gggtgaacgt gtggccacca gcatgcggac ggtaggcaag ctcccgcggc atcggcccct
2161 gagccgcact cagtcctcac cgctgccgca gagtccccag gccctgcagc agctggtcat
2221 gcaacaacag caccagcagt tcctggagaa gcagaagcag cagcagctac agctgggcaa
2281 gatcctcacc aagacagggg agctgcccag gcagcccacc acccaccctg aggagacaga
2341 ggaggagctg acggagcagc aggaggtctt gctgggggag ggagccctga ccatgccccg
2401 ggagggctcc acagagagtg agagcacaca ggaagacctg gaggaggagg acgaggaaga
2461 cgatggggag gaggaggagg attgcatcca ggttaaggac gaggagggcg agagtggtgc

2521 tgaggagggg cccgacttgg aggagcctgg tgctggatac aaaaaactgt tctcagatgc
2581 ccagccgctg cagcctttgc aggtgtacca ggcgcccctc agcctggcca ctgtgcccca
2641 ccaggccctg ggccgtaccc agtcctcccc tgctgcccct gggggcatga agagcccccc
2701 agaccagccc gtcaagcacc tcttcaccac aggtgtggtc tacgacacgt tcatgctaaa
2761 gcaccagtgc atgtgcggga acacacacgt gcaccctgag catgctggcc ggatccagag
2821 catctggtcc cggctgcagg agacaggcct gcttagcaag tgcgagcgga tccgaggtcg
2881 caaagccacg ctagatgaga tccagacagt gcactctgaa taccacaccc tgctctatgg
2941 gaccagtccc ctcaaccggc agaagctaga cagcaagaag ttgctcggcc ccatcagcca
3001 gaagatgtat gctgtgctgc cttgtggggg catcggggtg gacagtgaca ccgtgtggaa
3061 tgagatgcac tcctccagtg ctgtgcgcat ggcagtgggc tgcctgctgg agctggcctt
3121 caaggtggct gcaggagagc tcaagaatgg atttgccatc atccggcccc caggacacca
3181 cgccgaggaa tccacagcca tgggattctg cttcttcaac tctgtagcca tcaccgcaaa
3241 actcctacag cagaagttga acgtgggcaa ggtcctcatc gtggactggg acattcacca
3301 tggcaatggc acccagcagg cgttctacaa tgacccctct gtgctctaca tctctctgca
3361 tcgctatgac aacgggaact tctttccagg ctctggggct cctgaagagg ttggtggagg
3421 accaggcgtg gggtacaatg tgaacgtggc atggacagga ggtgtggacc cccccattgg
3481 agacgtggag taccttacag ccttcaggac agtggtgatg cccattgccc acgagttctc
3541 acctgatgtg gtcctagtct ccgccgggtt tgatgctgtt gaaggacatc tgtctcctct
3601 gggtggctac tctgtcaccg ccagatgttt tggccacttg accaggcagc tgatgaccct
3661 ggcagggggc cgggtggtgc tggccctgga gggaggccat gacttgaccg ccatctgtga
3721 tgcctctgag gcttgtgtct cggctctgct cagtgtagag ctgcagccct tggatgaggc
3781 agtcttgcag caaaagccca acatcaacgc agtggccacg ctagagaaag tcatcgagat
3841 ccagagcaaa cactggagct gtgtgcagaa gttcgccgct ggtctgggcc ggtccctgcg
3901 agaggcccaa gcaggtgaga ccgaggaggc cgagactgtg agcgccatgg ccttgctgtc
3961 ggtgggggcc gagcaggccc aggctgcggc agcccgggaa cacagcccca ggccggcaga
4021 ggagcccatg gagcaggagc ctgccctgct cgaggaacaa aaactaatat cagaggaaga
4081 tttggaacag aaactaatca gtgaagaaga tttagagcaa aagttaattt ccgaggagga

4141 cttatagtga gggcccgata tctcgacaat caacctctgg attacaaaat ttgtgaaaga
4201 ttgactggta ttcttaacta tgttgctcct tttacgctat gtggatacgc tgctttaatg
4261 cctttgtatc atgctattgc ttcccgtatg gctttcattt tctcctcctt gtataaatcc
4321 tggttgctgt ctctttatga ggagttgtgg cccgttgtca ggcaacgtgg cgtggtgtgc
4381 actgtgtttg ctgacgcaac ccccactggt tggggcattg ccaccacctg tcagctcctt
4441 tccgggactt tcgctttccc cctccctatt gccacggcgg aactcatcgc cgcctgcctt
4501 gcccgctgct ggacaggggc tcggctgttg ggcactgaca attccgtggt gttgtcgggg
4561 aagctgacgt cctttccatg gctgctcgcc tgtgttgcca cctggattct gcgcgggacg
4621 tccttctgct acgtcccttc ggccctcaat ccagcggacc ttccttcccg cggcctgctg
4681 ccggctctgc ggcctcttcc gcgtcttcgc cttcgccctc agacgagtcg gatctccctt
4741 tgggccgcct ccccgcctgg aaacgggggа ggctaactga aacacggaag gagacaatac
4801 cggaaggaac ccgcgctatg acggcaataa aaagacagaa taaaacgcac gggtgttggg
4861 tcgtttgttc ataaacgcgg ggttcggtcc cagggctggc actctgtcga taccccaccg
4921 agaccccatt ggggccaata cgcccgcgtt tcttcctttt ccccacccca ccccccaagt
4981 tcgggtgaag gcccagggct cgcagccaac gtcggggcgg caggccctgc catagcagat
5041 ctgcgcagct ggggctctag gggtatcccc cacgcgccct gtagcggcgc attaagcgcg
5101 gcgggtgtgg tggttacgcg cagcgtgacc gctacacttg ccagcgccct agcgcccgct
5161 cctttcgctt tcttcccttc ctttctcgcc acgttcgccg gctttccccg tcaagctcta
5221 aatcggggca tccctttagg gttccgattt agtgctttac ggcacctcga ccccaaaaaa
5281 cttgattagg gtgatggttc acgtagtggg ccatcgccct gatagacggt ttttcgccct
5341 ttgacgttgg agtccacgtt ctttaatagt ggactcttgt tccaaactgg aacaacactc
5401 aaccctatct cggtctattc ttttgattta taagggattt tggggatttc ggcctattgg
5461 ttaaaaaatg agctgattta acaaaaattt aacgcgaatt aattctgtgg aatgtgtgtc
5521 agttagggtg tggaaagtcc ccaggctccc cagcaggcag aagtatgcaa agcatgcatc
5581 tcaattagtc agcaaccagg tgtggaaagt ccccaggctc cccagcaggc agaagtatgc
5641 aaagcatgca tctcaattag tcagcaacca tagtcccgcc cctaactccg cccatcccgc
5701 ccctaactcc gcccagttcc gcccattctc cgccccatgg ctgactaatt ttttttattt

5761 atgcagaggc cgaggccgcc tctgcctctg agctattcca gaagtagtga ggaggctttt
5821 ttggaggcct aggcttttgc aaaaagctcc cgggagcttg tatatccatt ttcggatctg
5881 atcaagagac aggatgagga tcgtttcgca tgattgaaca agatggattg cacgcaggtt
5941 ctccggccgc ttgggtggag aggctattcg gctatgactg ggcacaacag acaatcggct
6001 gctctgatgc cgccgtgttc cggctgtcag cgcaggggcg cccggttctt tttgtcaaga
6061 ccgacctgtc cggtgccctg aatgaactgc aggacgaggc agcgcggcta tcgtggctgg
6121 ccacgacggg cgttccttgc gcagctgtgc tcgacgttgt cactgaagcg ggaagggact
6181 ggctgctatt gggcgaagtg ccggggcagg atctcctgtc atctcacctt gctcctgccg
6241 agaaagtatc catcatggct gatgcaatgc ggcggctgca tacgcttgat ccggctacct
6301 gcccattcga ccaccaagcg aaacatcgca tcgagcgagc acgtactcgg atggaagccg
6361 gtcttgtcga tcaggatgat ctggacgaag agcatcaggg gctcgcgcca gccgaactgt
6421 tcgccaggct caaggcgcgc atgcccgacg gcgaggatct cgtcgtgacc catggcgatg
6481 cctgcttgcc gaatatcatg gtggaaaatg gccgcttttc tggattcatc gactgtggcc
6541 ggctgggtgt ggcggaccgc tatcaggaca tagcgttggc tacccgtgat attgctgaag
6601 agcttggcgg cgaatgggct gaccgcttcc tcgtgcttta cggtatcgcc gctcccgatt
6661 cgcagcgcat cgccttctat cgccttcttg acgagttctt ctgagcggga ctctggggtt
6721 cgcgaaatga ccgaccaagc gacgcccaac ctgccatcac gagatttcga ttccaccgcc
6781 gccttctatg aaaggttggg cttcggaatc gttttccggg acgccggctg gatgatcctc
6841 cagcgcgggg atctcatgct ggagttcttc gcccacccca acttgtttat tgcagcttat
6901 aatggttaca aataaagcaa tagcatcaca aatttcacaa ataaagcatt tttttcactg
6961 cattctagtt gtggtttgtc caaactcatc aatgtatctt atcatgtctg tataccgtcg
7021 acctctagct agagcttggc gtaatcatgg tcatagctgt ttcctgtgtg aaattgttat
7081 ccgctcacaa ttccacacaa catacgagcc ggaagcataa agtgtaaagc ctggggtgcc
7141 taatgagtga gctaactcac attaattgcg ttgcgctcac tgcccgcttt ccagtcggga
7201 aacctgtcgt gccagctgca ttaatgaatc ggccaacgcg cggggagagg cggtttgcgt
7261 attgggcgct cttccgcttc ctcgctcact gactcgctgc gctcggtcgt tcggctgcgg
7321 cgagcggtat cagctcactc aaaggcggta atacggttat ccacagaatc aggggataac

7381 gcaggaaaga acatgtgagc aaaaggccag caaaaggcca ggaaccgtaa aaaggccgcg
7441 ttgctggcgt ttttccatag gctccgcccc cctgacgagc atcacaaaaa tcgacgctca
7501 agtcagaggt ggcgaaaccc gacaggacta taaagatacc aggcgtttcc ccctggaagc
7561 tccctcgtgc gctctcctgt tccgaccctg ccgcttaccg gatacctgtc cgcctttctc
7621 ccttcgggaa gcgtggcgct ttctcaatgc tcacgctgta ggtatctcag ttcggtgtag
7681 gtcgttcgct ccaagctggg ctgtgtgcac gaaccccccg ttcagcccga ccgctgcgcc
7741 ttatccggta actatcgtct tgagtccaac ccggtaagac acgacttatc gccactggca
7801 gcagccactg gtaacaggat tagcagagcg aggtatgtag gcggtgctac agagttcttg
7861 aagtggtggc ctaactacgg ctacactaga aggacagtat ttggtatctg cgctctgctg
7921 aagccagtta ccttcggaaa aagagttggt agctcttgat ccggcaaaca aaccaccgct
7981 ggtagcggtg gtttttttgt ttgcaagcag cagattacgc gcagaaaaaa aggatctcaa
8041 gaagatcctt tgatcttttc tacggggtct gacgctcagt ggaacgaaaa ctcacgttaa
8101 gggattttgg tcatgagatt atcaaaaagg atcttcacct agatcctttt aaattaaaaa
8161 tgaagtttta aatcaatcta aagtatatat gagtaaactt ggtctgacag ttaccaatgc
8221 ttaatcagtg aggcacctat ctcagcgatc tgtctatttc gttcatccat agttgcctga
8281 ctccccgtcg tgtagataac tacgatacgg gagggcttac catctggccc cagtgctgca
8341 atgataccgc gagacccacg ctcaccggct ccagatttat cagcaataaa ccagccagcc
8401 ggaagggccg agcgcagaag tggtcctgca actttatccg cctccatcca gtctattaat
8461 tgttgccggg aagctagagt aagtagttcg ccagttaata gtttgcgcaa cgttgttgcc
8521 attgctacag gcatcgtggt gtcacgctcg tcgtttggta tggcttcatt cagctccggt
8581 tcccaacgat caaggcgagt tacatgatcc cccatgttgt gcaaaaaagc ggttagctcc
8641 ttcggtcctc cgatcgttgt cagaagtaag ttggccgcag tgttatcact catggttatg
8701 gcagcactgc ataattctct tactgtcatg ccatccgtaa gatgcttttc tgtgactggt
8761 gagtactcaa ccaagtcatt ctgagaatag tgtatgcggc gaccgagttg ctcttgcccg
8821 gcgtcaatac gggataatac cgcgccacat agcagaactt taaaagtgct catcattgga
8881 aaacgttctt cggggcgaaa actctcaagg atcttaccgc tgttgagatc cagttcgatg
8941 taacccactc gtgcacccaa ctgatcttca gcatctttta ctttcaccag cgtttctggg

9001 tgagcaaaaa caggaaggca aaatgccgca aaaaagggaa taagggcgac
acggaaatgt
9061 tgaatactca tactcttcct ttttcaatat tattgaagca tttatcaggg
ttattgtctc
9121 atgagcggat acatatttga atgtatttag aaaaataaac aaataggggt
tccgcgcaca
9181 tttccccgaa aagtgccacc tgacgtcgac ggatcgggag atctcccgat
cccctatggt
9241 cgactctcag tacaatctgc tctgatgccg catagttaag ccagtatctg
ctccctgctt
9301 gtgtgttgga ggtcgctgag tagtgcgcga gcaaaattta agctacaaca
aggcaaggct
9361 tgaccgacaa ttgcatgaag aatctgctta gg
//
