# Supplementary material for: Identification of Critical Phosphorylation Sites Enhancing Kinase Activity With a Bimodal Fusion Framework
Source: Mol Cell Proteomics. 2024 Nov 30;24(1):100889. doi: 10.1016/j.mcpro.2024.100889 (PMC11774822; doi:10.1016/j.mcpro.2024.100889)
Supplement: Supplemental Data 2 [file mmc4.pdf]

```
LOCUS       Exported                8705 bp ds-DNA    circular SYN
07-3月-2024
DEFINITION  .
ACCESSION   .
VERSION     .
KEYWORDS    Untitled 56
SOURCE      synthetic DNA construct
  ORGANISM  synthetic DNA construct
REFERENCE   1  (bases 1 to 8705)
  AUTHORS   111111111
  TITLE     Direct Submission
  JOURNAL   Exported 2024年3月7日 from SnapGene 2.3.2
            http://www.snapgene.com
FEATURES             Location/Qualifiers
     source          1..8705
                     /organism="synthetic DNA construct"
                     /mol_type="other DNA"
     enhancer        50..429
                     /note="CMV enhancer"
                     /note="human cytomegalovirus immediate early
enhancer"
     promoter        430..633
                     /note="CMV promoter"
                     /note="human cytomegalovirus (CMV) immediate
early
                     promoter"
     CDS             3392..3457
                     /codon_start=1
                     /product="three tandem FLAG(R) epitope tags,
followed by an
                     enterokinase cleavage site"
                     /note="3xFLAG"
                     /translation="DYKDHDGDYKDHDIDYKDDDDK"
     misc_feature    3481..4069
                     /note="WPRE"
                     /note="woodchuck hepatitis virus
posttranscriptional
                     regulatory element"
     CDS             complement(3952..3963)
                     /codon_start=1
                     /product="Factor Xa recognition and cleavage
site"
                     /note="Factor Xa site"
                     /translation="IEGR"
     polyA_signal    4135..4183
                     /note="HSV TK poly(A) signal"
                     /note="herpesvirus thymidine kinase
polyadenylation signal"
     rep_origin      4385..4813
                     /direction=RIGHT
                     /note="f1 ori"
                     /note="f1 bacteriophage origin of replication;
arrow
                     indicates direction of (+) strand synthesis"
```

```
     promoter        4827..5156
                     /note="SV40 promoter"
                     /note="SV40 enhancer and early promoter"
     rep_origin      5007..5142
                     /note="SV40 ori"
                     /note="SV40 origin of replication"
     CDS             5223..6017
                     /codon_start=1
                     /gene="aph(3')-II (or nptII)"
                     /product="aminoglycoside phosphotransferase
from Tn5"
                     /note="NeoR/KanR"
                     /note="confers resistance to neomycin,
kanamycin, and G418
                     (Geneticin(R))"
                     /
translation="MIEQDGLHAGSPAAWVERLFGYDWAQQTIGCSDAAVFRLSAQGRP

VLFVKTDLSGALNELQDEAARLSWLATTGVPCAAVLDVVTEAGRDWLLLGEVPGQDLLS

SHLAPAEKVSIMADAMRRLHTLDPATCPFDHQAKHRIERARTRMEAGLVDQDDLDEEHQ

GLAPAELFARLKARMPDGEDLVVTHGDACLPNIMVENGRFSGFIDCGRLGVADRYQDIA
                     LATRDIAEELGGEWADRFLVLYGIAAPDSQRIAFYRLLDEFF"
     polyA_signal    6193..6314
                     /note="SV40 poly(A) signal"
                     /note="SV40 polyadenylation signal"
     primer_bind     complement(6363..6379)
                     /note="M13 rev"
                     /note="common sequencing primer, one of
multiple similar
                     variants"
     protein_bind    6387..6403
                     /bound_moiety="lac repressor encoded by lacI"
                     /note="lac operator"
                     /note="The lac repressor binds to the lac
operator to
                     inhibit transcription in E. coli. This
inhibition can be
                     relieved by adding lactose or
                     isopropyl-beta-D-thiogalactopyranoside (IPTG)."
     promoter        complement(6411..6441)
                     /note="lac promoter"
                     /note="promoter for the E. coli lac operon"
     protein_bind    6456..6477
                     /bound_moiety="E. coli catabolite activator
protein"
                     /note="CAP binding site"
                     /note="CAP binding activates transcription in
the presence
                     of cAMP."
     rep_origin      complement(6765..7353)
                     /direction=LEFT
                     /note="ori"
```

```
                     /note="high-copy-number ColE1/pMB1/pBR322/pUC
origin of
                     replication"
     CDS             complement(7524..8384)
                     /codon_start=1
                     /gene="bla"
                     /product="beta-lactamase"
                     /note="AmpR"
                     /note="confers resistance to ampicillin,
carbenicillin, and
                     related antibiotics"
                     /
translation="MSIQHFRVALIPFFAAFCLPVFAHPETLVKVKDAEDQLGARVGYI

ELDLNSGKILESFRPEERFPMMSTFKVLLCGAVLSRIDAGQEQLGRRIHYSQNDLVEYS

PVTEKHLTDGMTVRELCSAAITMSDNTAANLLLTTIGGPKELTAFLHNMGDHVTRLDRW

EPELNEAIPNDERDTTMPVAMATTLRKLLTGELLTLASRQQLIDWMEADKVAGPLLRSA

LPAGWFIADKSGAGERGSRGIIAALGPDGKPSRIVVIYTTGSQATMDERNRQIAEIGAS
                     LIKHW"
     promoter        complement(8385..8489)
                     /gene="bla"
                     /note="AmpR promoter"
ORIGIN
        1 gttaggcgtt ttgcgctgct tcgcgatgta cgggccagat atacgcgttg
acattgatta
       61 ttgactagtt attaatagta atcaattacg gggtcattag ttcatagccc
atatatggag
      121 ttccgcgtta cataacttac ggtaaatggc ccgcctggct gaccgcccaa
cgacccccgc
      181 ccattgacgt caataatgac gtatgttccc atagtaacgc caatagggac
tttccattga
      241 cgtcaatggg tggagtattt acggtaaact gcccacttgg cagtacatca
agtgtatcat
      301 atgccaagta cgccccctat tgacgtcaat gacggtaaat ggcccgcctg
gcattatgcc
      361 cagtacatga ccttatggga ctttcctact tggcagtaca tctacgtatt
agtcatcgct
      421 attaccatgg tgatgcggtt ttggcagtac atcaatgggc gtggatagcg
gtttgactca
      481 cggggatttc caagtctcca ccccattgac gtcaatggga gtttgttttg
gcaccaaaat
      541 caacgggact ttccaaaatg tcgtaacaac tccgccccat tgacgcaaat
gggcggtagg
      601 cgtgtacggt gggaggtcta tataagcaga gctctggcta gcgtttaaac
ttaagcttgg
      661 taccgagctc ggatccgcca ccatgtctgc aaataattcc cctccatcag
cccagaagtc
      721 tgtattaccc acagctattc ctgctgtgct tccagctgct tctccgtgtt
caagtcctaa
      781 gacgggactc tctgcccgac tctctaatgg aagcttcagt gcaccatcac
tcaccaactc
```

841 cagaggctca gtgcatacag tttcatttct actgcaaatt ggcctcacac
gggagagtgt
901 taccattgaa gcccaggaac tgtctttatc tgctgtcaag gatcttgtgt
gctccatagt
961 ttatcaaaag tttccagagt gtggattctt tggcatgtat gacaaaattc
ttctctttcg
1021 ccatgacatg aactcagaaa acattttgca gctgattacc tcagcagatg
aaatacatga
1081 aggagaccta gtggaagtgg ttctttcagc tttagccaca gtagaagact
tccagattcg
1141 tccacatact ctctatgtac attcttacaa agctcctact ttctgtgatt
actgtggtga
1201 gatgctctgg ggattggtac gtcaaggact gaaatgtgaa ggctgtggat
taaattacca
1261 taaacgatgt gccttcaaga ttccaaataa ctgtagtgga gtaagaaaga
gacgtctgtc
1321 aaatgtatct ttaccaggac ccggcctctc agttccaaga cccctacagc
ctgaatatgt
1381 agcccttccc agtgaagagt cacatgtcca ccaggaacca agtaagagaa
ttccttcttg
1441 gagtggtcgc ccaatctgga tggaaaagat ggtaatgtgc agagtgaaag
ttccacacac
1501 atttgctgtt cactcttaca cccgtcccac gatatgtcag tactgcaagc
ggttactgaa
1561 aggcctcttt cgccaaggaa tgcagtgtaa agattgcaaa ttcaactgcc
ataaacgctg
1621 tgcatcaaaa gtaccaagag actgccttgg agaggttact ttcaatggag
aaccttccag
1681 tctgggaaca gatacagata taccaatgga tattgacaat aatgacataa
atagtgatag
1741 tagtcggggt ttggatgaca cagaagagcc atcacccccca gaagataaga
tgttcttctt
1801 ggatccatct gatctcgatg tggaaagaga tgaagaagcc gttaaaacaa
tcagtccatc
1861 aacaagcaat aatattccgc taatgagggt tgtacaatcc atcaagcaca
caaagaggaa
1921 gagcagcaca atggtgaagg aagggtggat ggtccattac accagcaggg
ataacctgag
1981 aaagaggcat tattggagac ttgacagcaa atgtctaaca ttatttcaga
atgaatctgg
2041 atcaaagtat tataaggaaa ttccactttc agaaattctc cgcatatctt
caccacgaga
2101 tttcacaaac atttcacaag gcagcaatcc acactgtttt gaaatcatta
ctgatactat
2161 ggtatacttc gttggtgaga acaatgggga cagctctcat aatcctgttc
ttgctgccac
2221 tggagttgga cttgatgtag cacagagctg ggaaaaagca attcgccaag
ccctcatgcc
2281 tgttactcct caagcaagtg tttgcacttc tccagggcaa gggaaagatc
acaaagattt
2341 gtctacaagt atctctgtat ctaattgtca gattcaggag aatgtggata
tcagtactgt
2401 ttaccagatc tttgcagatg aggtgcttgg ttcaggccag tttggcatcg
tttatggagg

2461 aaaacataga aagactggga gggatgtggc tattaaagta attgataaga
tgagattccc
2521 cacaaaacaa gaaagtcaac tccgtaatga agtggctatt ttacagaatt
tgcaccatcc
2581 tgggattgta aacctggaat gtatgtttga aaccccagaa cgagtctttg
tagtaatgga
2641 aaagctgcat ggagatatgt tggaaatgat tctatccagt gagaaaagtc
ggcttccaga
2701 acgaattact aaattcatgg tcacacagat acttgttgct ttgaggaatc
tgcattttaa
2761 gaatattgtg cactgtgatt taaagccaga aaatgtgctg cttgcatcag
cagagccatt
2821 tcctcaggtg aagctgtgtg actttggatt tgcacgcatc attggtgaaa
agtcattcag
2881 gagatctgtg gtaggaactc cagcatactt agcccctgaa gttctccgga
gcaaaggtta
2941 caaccgttcc ctagatatgt ggtcagtggg agttatcatc tatgtgagcc
tcagtggcac
3001 atttcctttt aatgaggatg aagatataaa tgaccaaatc caaaatgctg
catttatgta
3061 cccaccaaat ccatggagag aaatttctgg tgaagcaatt gatctgataa
acaatctgct
3121 tcaagtgaag atgagaaaac gttacagtgt tgacaaatct cttagtcatc
cctggctaca
3181 ggactatcag acttggcttg accttagaga atttgaaact cgcattggag
aacgttacat
3241 tacacatgaa agtgatgatg ctcgctggga aatacatgca tacacacata
accttgtata
3301 cccaaagcac ttcattatgg ctcctaatcc agatgatatg gaagaagatc
ctgaattctg
3361 cagatatcca gcacagtggc ggccgctcga ggactacaaa gaccatgacg
gtgattataa
3421 agatcatgac atcgactaca aggatgacga tgacaagtag tgagggcccg
atatctcgac
3481 aatcaacctc tggattacaa aatttgtgaa agattgactg gtattcttaa
ctatgttgct
3541 ccttttacgc tatgtggata cgctgcttta atgcctttgt atcatgctat
tgcttcccgt
3601 atggctttca ttttctcctc cttgtataaa tcctggttgc tgtctcttta
tgaggagttg
3661 tggcccgttg tcaggcaacg tggcgtggtg tgcactgtgt ttgctgacgc
aacccccact
3721 ggttggggca ttgccaccac ctgtcagctc ctttccggga ctttcgcttt
ccccctccct
3781 attgccacgg cggaactcat cgccgcctgc cttgcccgct gctggacagg
ggctcggctg
3841 ttgggcactg acaattccgt ggtgttgtcg gggaagctga cgtcctttcc
atggctgctc
3901 gcctgtgttg ccacctggat tctgcgcggg acgtccttct gctacgtccc
ttcggccctc
3961 aatccagcgg accttccttc ccgcggcctg ctgccggctc tgcggcctct
tccgcgtctt
4021 cgccttcgcc ctcagacgag tcggatctcc ctttgggccg cctccccgcc
tggaaacggg

4081 ggaggctaac tgaaacacgg aaggagacaa taccggaagg aacccgcgct atgacggcaa
4141 taaaaagaca gaataaaacg cacgggtgtt gggtcgtttg ttcataaacg cggggttcgg
4201 tcccagggct ggcactctgt cgatacccca ccgagacccc attggggcca atacgcccgc
4261 gtttcttcct tttccccacc ccacccccca agttcgggtg aaggcccagg gctcgcagcc
4321 aacgtcgggg cggcaggccc tgccatagca gatctgcgca gctggggctc taggggggtat
4381 ccccacgcgc cctgtagcgg cgcattaagc gcggcgggtg tggtggttac gcgcagcgtg
4441 accgctacac ttgccagcgc cctagcgccc gctcctttcg ctttcttccc ttcctttctc
4501 gccacgttcg ccggctttcc ccgtcaagct ctaaatcggg gcatcccttt agggttccga
4561 tttagtgctt tacggcacct cgaccccaaa aaacttgatt agggtgatgg ttcacgtagt
4621 gggccatcgc cctgatagac ggtttttcgc cctttgacgt tggagtccac gttctttaat
4681 agtggactct tgttccaaac tggaacaaca ctcaacccta tctcggtcta ttcttttgat
4741 ttataaggga ttttggggat ttcggcctat tggttaaaaa atgagctgat ttaacaaaaa
4801 tttaacgcga attaattctg tggaatgtgt gtcagttagg gtgtggaaag tccccaggct
4861 ccccagcagg cagaagtatg caaagcatgc atctcaatta gtcagcaacc aggtgtggaa
4921 agtccccagg ctccccagca ggcagaagta tgcaaagcat gcatctcaat tagtcagcaa
4981 ccatagtccc gcccctaact ccgcccatcc cgcccctaac tccgcccagt tccgcccatt
5041 ctccgcccca tggctgacta atttttttta tttatgcaga ggccgaggcc gcctctgcct
5101 ctgagctatt ccagaagtag tgaggaggct tttttggagg cctaggcttt tgcaaaaagc
5161 tcccgggagc ttgtatatcc attttcggat ctgatcaaga gacaggatga ggatcgtttc
5221 gcatgattga acaagatgga ttgcacgcag gttctccggc cgcttgggtg gagaggctat
5281 tcggctatga ctgggcacaa cagacaatcg gctgctctga tgccgccgtg ttccggctgt
5341 cagcgcaggg gcgcccggtt ctttttgtca agaccgacct gtccggtgcc ctgaatgaac
5401 tgcaggacga ggcagcgcgg ctatcgtggc tggccacgac gggcgttcct tgcgcagctg
5461 tgctcgacgt tgtcactgaa gcgggaaggg actggctgct attgggcgaa gtgccggggc
5521 aggatctcct gtcatctcac cttgctcctg ccgagaaagt atccatcatg gctgatgcaa
5581 tgcggcggct gcatacgctt gatccggcta cctgcccatt cgaccaccaa gcgaaacatc
5641 gcatcgagcg agcacgtact cggatggaag ccggtcttgt cgatcaggat gatctggacg

5701 aagagcatca ggggctcgcg ccagccgaac tgttcgccag gctcaaggcg
cgcatgcccg
5761 acggcgagga tctcgtcgtg acccatggcg atgcctgctt gccgaatatc
atggtggaaa
5821 atggccgctt ttctggattc atcgactgtg gccggctggg tgtggcggac
cgctatcagg
5881 acatagcgtt ggctacccgt gatattgctg aagagcttgg cggcgaatgg
gctgaccgct
5941 tcctcgtgct ttacggtatc gccgctcccg attcgcagcg catcgccttc
tatcgccttc
6001 ttgacgagtt cttctgagcg ggactctggg gttcgcgaaa tgaccgacca
agcgacgccc
6061 aacctgccat cacgagattt cgattccacc gccgccttct atgaaaggtt
gggcttcgga
6121 atcgttttcc gggacgccgg ctggatgatc ctccagcgcg gggatctcat
gctggagttc
6181 ttcgcccacc ccaacttgtt tattgcagct tataatggtt acaaataaag
caatagcatc
6241 acaaatttca caaataaagc atttttttca ctgcattcta gttgtggttt
gtccaaactc
6301 atcaatgtat cttatcatgt ctgtataccg tcgacctcta gctagagctt
ggcgtaatca
6361 tggtcatagc tgtttcctgt gtgaaattgt tatccgctca caattccaca
caacatacga
6421 gccggaagca taaagtgtaa agcctggggt gcctaatgag tgagctaact
cacattaatt
6481 gcgttgcgct cactgcccgc tttccagtcg ggaaacctgt cgtgccagct
gcattaatga
6541 atcggccaac gcgcggggag aggcggtttg cgtattgggc gctcttccgc
ttcctcgctc
6601 actgactcgc tgcgctcggt cgttcggctg cggcgagcgg tatcagctca
ctcaaaggcg
6661 gtaatacggt tatccacaga atcaggggat aacgcaggaa agaacatgtg
agcaaaaggc
6721 cagcaaaagg ccaggaaccg taaaaaggcc gcgttgctgg cgtttttcca
taggctccgc
6781 ccccctgacg agcatcacaa aaatcgacgc tcaagtcaga ggtggcgaaa
cccgacagga
6841 ctataaagat accaggcgtt tccccctgga agctccctcg tgcgctctcc
tgttccgacc
6901 ctgccgctta ccggatacct gtccgccttt ctcccttcgg gaagcgtggc
gctttctcaa
6961 tgctcacgct gtaggtatct cagttcggtg taggtcgttc gctccaagct
gggctgtgtg
7021 cacgaacccc ccgttcagcc cgaccgctgc gccttatccg gtaactatcg
tcttgagtcc
7081 aacccggtaa gacacgactt atcgccactg gcagcagcca ctggtaacag
gattagcaga
7141 gcgaggtatg taggcggtgc tacagagttc ttgaagtggt ggcctaacta
cggctacact
7201 agaaggacag tatttggtat ctgcgctctg ctgaagccag ttaccttcgg
aaaaagagtt
7261 ggtagctctt gatccggcaa acaaaccacc gctggtagcg gtggtttttt
tgtttgcaag

```
     7321 cagcagatta cgcgcagaaa aaaaggatct caagaagatc ctttgatctt
ttctacgggg
     7381 tctgacgctc agtggaacga aaactcacgt taagggattt tggtcatgag
attatcaaaa
     7441 aggatcttca cctagatcct tttaaattaa aaatgaagtt ttaaatcaat
ctaaagtata
     7501 tatgagtaaa cttggtctga cagttaccaa tgcttaatca gtgaggcacc
tatctcagcg
     7561 atctgtctat ttcgttcatc catagttgcc tgactccccg tcgtgtagat
aactacgata
     7621 cgggagggct taccatctgg ccccagtgct gcaatgatac cgcgagaccc
acgctcaccg
     7681 gctccagatt tatcagcaat aaaccagcca gccggaaggg ccgagcgcag
aagtggtcct
     7741 gcaactttat ccgcctccat ccagtctatt aattgttgcc gggaagctag
agtaagtagt
     7801 tcgccagtta atagtttgcg caacgttgtt gccattgcta caggcatcgt
ggtgtcacgc
     7861 tcgtcgtttg gtatggcttc attcagctcc ggttcccaac gatcaaggcg
agttacatga
     7921 tcccccatgt tgtgcaaaaa agcggttagc tccttcggtc ctccgatcgt
tgtcagaagt
     7981 aagttggccg cagtgttatc actcatggtt atggcagcac tgcataattc
tcttactgtc
     8041 atgccatccg taagatgctt ttctgtgact ggtgagtact caaccaagtc
attctgagaa
     8101 tagtgtatgc ggcgaccgag ttgctcttgc ccggcgtcaa tacgggataa
taccgcgcca
     8161 catagcagaa ctttaaaagt gctcatcatt ggaaaacgtt cttcggggcg
aaaactctca
     8221 aggatcttac cgctgttgag atccagttcg atgtaaccca ctcgtgcacc
caactgatct
     8281 tcagcatctt ttactttcac cagcgtttct gggtgagcaa aaacaggaag
gcaaaatgcc
     8341 gcaaaaaagg gaataagggc gacacggaaa tgttgaatac tcatactctt
cctttttcaa
     8401 tattattgaa gcatttatca gggttattgt ctcatgagcg gatacatatt
tgaatgtatt
     8461 tagaaaaata aacaaatagg ggttccgcgc acatttcccc gaaaagtgcc
acctgacgtc
     8521 gacggatcgg gagatctccc gatcccctat ggtcgactct cagtacaatc
tgctctgatg
     8581 ccgcatagtt aagccagtat ctgctccctg cttgtgtgtt ggaggtcgct
gagtagtgcg
     8641 cgagcaaaat ttaagctaca acaaggcaag gcttgaccga caattgcatg
aagaatctgc
     8701 ttagg
//
```
