# Supplementary material for: Identification of Critical Phosphorylation Sites Enhancing Kinase Activity With a Bimodal Fusion Framework
Source: Mol Cell Proteomics. 2024 Nov 30;24(1):100889. doi: 10.1016/j.mcpro.2024.100889 (PMC11774822; doi:10.1016/j.mcpro.2024.100889)
Supplement: Supplemental Data 6 [file mmc8.pdf]

```
LOCUS       Exported                7421 bp ds-DNA    circular SYN
24-1月-2024
DEFINITION  .
ACCESSION   .
VERSION     .
KEYWORDS    Untitled 29
SOURCE      synthetic DNA construct
  ORGANISM  synthetic DNA construct
REFERENCE   1  (bases 1 to 7421)
  AUTHORS   111111
  TITLE     Direct Submission
  JOURNAL   Exported 2024年1月24日 from SnapGene 2.3.2
            http://www.snapgene.com
FEATURES             Location/Qualifiers
     source          1..7421
                     /organism="synthetic DNA construct"
                     /mol_type="other DNA"
     enhancer        235..614
                     /note="CMV enhancer"
                     /note="human cytomegalovirus immediate early
enhancer"
     promoter        615..818
                     /note="CMV promoter"
                     /note="human cytomegalovirus (CMV) immediate
early
                     promoter"
     promoter        863..881
                     /note="T7 promoter"
                     /note="promoter for bacteriophage T7 RNA
polymerase"
     misc_feature    2211..2715
                     /note="pcDNA3.1(+)-human PRKG1-flag-T517A"
     CDS             2960..2983
                     /codon_start=1
                     /product="FLAG(R) epitope tag, followed by an
enterokinase
                     cleavage site"
                     /note="FLAG"
                     /translation="DYKDDDDK"
     polyA_signal    3021..3245
                     /note="bGH poly(A) signal"
                     /note="bovine growth hormone polyadenylation
signal"
     rep_origin      3291..3719
                     /direction=RIGHT
                     /note="f1 ori"
                     /note="f1 bacteriophage origin of replication;
arrow
                     indicates direction of (+) strand synthesis"
     promoter        3733..4062
                     /note="SV40 promoter"
                     /note="SV40 enhancer and early promoter"
     rep_origin      3913..4048
                     /note="SV40 ori"
```

```
                     /note="SV40 origin of replication"
     CDS             4129..4923
                     /codon_start=1
                     /gene="aph(3')-II (or nptII)"
                     /product="aminoglycoside phosphotransferase
from Tn5"
                     /note="NeoR/KanR"
                     /note="confers resistance to neomycin,
kanamycin, and G418
                     (Geneticin(R))"
                     /
translation="MIEQDGLHAGSPAAWVERLFGYDWAQQTIGCSDAAVFRLSAQGRP

VLFVKTDLSGALNELQDEAARLSWLATTGVPCAAVLDVVTEAGRDWLLLGEVPGQDLLS

SHLAPAEKVSIMADAMRRLHTLDPATCPFDHQAKHRIERARTRMEAGLVDQDDLDEEHQ

GLAPAELFARLKARMPDGEDLVVTHGDACLPNIMVENGRFSGFIDCGRLGVADRYQDIA
                     LATRDIAEELGGEWADRFLVLYGIAAPDSQRIAFYRLLDEFF"
     polyA_signal    5097..5218
                     /note="SV40 poly(A) signal"
                     /note="SV40 polyadenylation signal"
     primer_bind     complement(5267..5283)
                     /note="M13 rev"
                     /note="common sequencing primer, one of
multiple similar
                     variants"
     protein_bind    5291..5307
                     /bound_moiety="lac repressor encoded by lacI"
                     /note="lac operator"
                     /note="The lac repressor binds to the lac
operator to
                     inhibit transcription in E. coli. This
inhibition can be
                     relieved by adding lactose or
                     isopropyl-beta-D-thiogalactopyranoside (IPTG)."
     promoter        complement(5315..5345)
                     /note="lac promoter"
                     /note="promoter for the E. coli lac operon"
     protein_bind    5360..5381
                     /bound_moiety="E. coli catabolite activator
protein"
                     /note="CAP binding site"
                     /note="CAP binding activates transcription in
the presence
                     of cAMP."
     rep_origin      complement(5669..6254)
                     /direction=LEFT
                     /note="ori"
                     /note="high-copy-number ColE1/pMB1/pBR322/pUC
origin of
                     replication"
     CDS             complement(6425..7285)
                     /codon_start=1
```

```
                     /gene="bla"
                     /product="beta-lactamase"
                     /note="AmpR"
                     /note="confers resistance to ampicillin,
carbenicillin, and
                     related antibiotics"
                     /
translation="MSIQHFRVALIPFFAAFCLPVFAHPETLVKVKDAEDQLGARVGYI

ELDLNSGKILESFRPEERFPMMSTFKVLLCGAVLSRIDAGQEQLGRRIHYSQNDLVEYS

PVTEKHLTDGMTVRELCSAAITMSDNTAANLLLTTIGGPKELTAFLHNMGDHVTRLDRW

EPELNEAIPNDERDTTMPVAMATTLRKLLTGELLTLASRQQLIDWMEADKVAGPLLRSA

LPAGWFIADKSGAGERGSRGIIAALGPDGKPSRIVVIYTTGSQATMDERNRQIAEIGAS
                     LIKHW"
     promoter        complement(7286..7390)
                     /gene="bla"
                     /note="AmpR promoter"
ORIGIN
        1 gacggatcgg gagatctccc gatcccctat ggtgcactct cagtacaatc
tgctctgatg
       61 ccgcatagtt aagccagtat ctgctccctg cttgtgtgtt ggaggtcgct
gagtagtgcg
      121 cgagcaaaat ttaagctaca acaaggcaag gcttgaccga caattgcatg
aagaatctgc
      181 ttagggttag gcgttttgcg ctgcttcgcg atgtacgggc cagatatacg
cgttgacatt
      241 gattattgac tagttattaa tagtaatcaa ttacggggtc attagttcat
agcccatata
      301 tggagttccg cgttacataa cttacggtaa atggcccgcc tggctgaccg
cccaacgacc
      361 cccgcccatt gacgtcaata atgacgtatg ttcccatagt aacgccaata
gggactttcc
      421 attgacgtca atgggtggag tatttacggt aaactgccca cttggcagta
catcaagtgt
      481 atcatatgcc aagtacgccc cctattgacg tcaatgacgg taaatggccc
gcctggcatt
      541 atgcccagta catgacctta tgggactttc ctacttggca gtacatctac
gtattagtca
      601 tcgctattac catggtgatg cggttttggc agtacatcaa tgggcgtgga
tagcggtttg
      661 actcacgggg atttccaagt ctccacccca ttgacgtcaa tgggagtttg
ttttggcacc
      721 aaaatcaacg ggactttcca aaatgtcgta acaactccgc cccattgacg
caaatgggcg
      781 gtaggcgtgt acggtgggag gtctatataa gcagagctct ctggctaact
agagaaccca
      841 ctgcttactg gcttatcgaa attaatacga ctcactatag ggagacccaa
gctggctagc
      901 gtttaaactt aagcttggta ccgagctcgg atccgccacc atgagcgagc
tagaggaaga
      961 ctttgccaag attctcatgc tcaaggagga gaggatcaaa gagctggaga
```

agcggctgtc
1021 agagaaggag gaagaaattc aggagctgaa gaggaaactc cacaaatgcc agtcggtgct
1081 cccagtgccc tcgacccaca tcggcccccg gaccacccgg gcgcagggca tctcggccga
1141 gccgcagacg tacaggtcct tccacgacct ccgacaggca ttccggaagt tcaccaagtc
1201 cgaaaggtcc aaggatctta taaaggaagc tatccttgac aatgacttta tgaagaactt
1261 ggagctgtcg cagatccagg agattgtgga ttgtatgtac ccggtggagt atggcaagga
1321 cagttgcatc atcaaagaag gagacgtggg gtcactggtg tatgtcatgg aagatggtaa
1381 ggttgaagtt acaaaagaag gtgtgaagtt gtgtaccatg ggtccaggaa aagtgtttgg
1441 ggaattggct attctttaca actgtacccg gacagcgacc gtcaagactc ttgtaaatgt
1501 aaaactctgg gccattgatc gacaatgttt tcaaacaata atgatgagga caggactcat
1561 caagcatacc gagtatatgg aatttttaaa aagcgttcca acattccaga gccttcctga
1621 agagatcctc agcaagcttg ctgatgtcct tgaagagacc cactatgaaa atggagaata
1681 tattatcagg caaggtgcaa gaggggacac cttctttatc atcagcaaag gaacggtaaa
1741 tgtcactcgt gaagactcac cgagtgaaga cccagtcttt cttagaactt taggaaaagg
1801 agactggttt ggagagaaag ccttgcaggg ggaagatgtg agaacagcaa acgtaattgc
1861 tgcagaagct gtaacctgcc ttgtgattga cagagactct tttaaacatt tgattggagg
1921 gctggatgat gtttctaata aagcatatga agatgcagaa gctaaagcaa aatatgaagc
1981 tgaagcggct ttcttcgcca acctgaagct gtctgatttc aacatcattg ataccttgg
2041 agttggaggt ttcggacgag tagaactggt ccagttgaaa agtgaagaat ccaaaacgtt
2101 tgcaatgaag attctcaaga aacgtcacat tgtggacaca agacagcagg agcacatccg
2161 ctcagagaag cagatcatgc agggggctca ttccgatttc atagtgagac tgtacagaac
2221 atttaaggac agcaaatatt tgtatatgtt gatggaagct tgtctaggtg gagagctctg
2281 gaccattctc agggatagag gttcgtttga agattctaca accagatttt acacagcatg
2341 tgtggtagaa gcttttgcct atctgcattc caaaggaatc atttacaggg acctcaagcc
2401 agaaaatctc atcctagatc accgaggtta tgccaaactg gttgattttg gctttgcaaa
2461 gaaaatagga tttggaaaga aaacatgggc attttgtggg actccagagt atgtagcccc
2521 agagatcatc ctgaacaaag gccatgacat ttcagccgac tactggtcac tgggaatcct
2581 aatgtatgaa ctcctgactg gcagcccacc tttctcaggc ccagatccta

tgaaaaccta
2641 taacatcata ttgaggggga ttgacatgat agaatttcca aagaagattg ccaaaaatgc
2701 tgctaattta attaaaaaac tatgcaggga caatccatca gaaagattag ggaatttgaa
2761 aaatggagta aaagacattc aaaagcacaa atggtttgag ggctttaact gggaaggctt
2821 aagaaaaggt accttgacac ctcctataat accaagtgtt gcatcaccca cagacacaag
2881 taattttgac agtttccctg aggacaacga tgaaccacca cctgatgaca actcaggatg
2941 ggatatagac ttcctcgagg attacaagga tgacgacgat aagtagtgag ggcccgttta
3001 aacccgctga tcagcctcga ctgtgccttc tagttgccag ccatctgttg tttgcccctc
3061 ccccgtgcct tccttgaccc tggaaggtgc cactcccact gtcctttcct aataaaatga
3121 ggaaattgca tcgcattgtc tgagtaggtg tcattctatt ctggggggtg gggtggggca
3181 ggacagcaag ggggaggatt gggaagacaa tagcaggcat gctggggatg cggtgggctc
3241 tatggcttct gaggcggaaa gaaccagctg gggctctagg gggtatcccc acgcgccctg
3301 tagcggcgca ttaagcgcgg cgggtgtggt ggttacgcgc agcgtgaccg ctacacttgc
3361 cagcgcccta gcgcccgctc ctttcgcttt cttcccttcc tttctcgcca cgttcgccgg
3421 ctttccccgt caagctctaa atcgggggct ccctttaggg ttccgattta gtgctttacg
3481 gcacctcgac cccaaaaaac ttgattaggg tgatggttca cgtagtgggc catcgccctg
3541 atagacggtt tttcgccctt tgacgttgga gtccacgttc tttaatagtg gactcttgtt
3601 ccaaactgga acaacactca accctatctc ggtctattct tttgatttat aagggatttt
3661 gccgatttcg gcctattggt taaaaaatga gctgatttaa caaaaattta acgcgaatta
3721 attctgtgga atgtgtgtca gttagggtgt ggaaagtccc caggctcccc agcaggcaga
3781 agtatgcaaa gcatgcatct caattagtca gcaaccaggt gtggaaagtc cccaggctcc
3841 ccagcaggca gaagtatgca aagcatgcat ctcaattagt cagcaaccat agtcccgccc
3901 ctaactccgc ccatcccgcc cctaactccg cccagttccg cccattctcc gccccatggc
3961 tgactaattt tttttattta tgcagaggcc gaggccgcct ctgcctctga gctattccag
4021 aagtagtgag gaggcttttt tggaggccta ggcttttgca aaaagctccc gggagcttgt
4081 atatccattt tcggatctga tcaagagaca ggatgaggat cgtttcgcat gattgaacaa
4141 gatggattgc acgcaggttc tccggccgct tgggtggaga ggctattcgg ctatgactgg
4201 gcacaacaga caatcggctg ctctgatgcc gccgtgttcc ggctgtcagc

gcaggggcgc
4261 ccggttcttt ttgtcaagac cgacctgtcc ggtgccctga atgaactgca ggacgaggca
4321 gcgcggctat cgtggctggc cacgacgggc gttccttgcg cagctgtgct cgacgttgtc
4381 actgaagcgg gaagggactg gctgctattg ggcgaagtgc cggggcagga tctcctgtca
4441 tctcaccttg ctcctgccga gaaagtatcc atcatggctg atgcaatgcg gcggctgcat
4501 acgcttgatc cggctacctg cccattcgac caccaagcga aacatcgcat cgagcgagca
4561 cgtactcgga tggaagccgg tcttgtcgat caggatgatc tggacgaaga gcatcagggg
4621 ctcgcgccag ccgaactgtt cgccaggctc aaggcgcgca tgcccgacgg cgaggatctc
4681 gtcgtgaccc atggcgatgc ctgcttgccg aatatcatgg tggaaaatgg ccgcttttct
4741 ggattcatcg actgtggccg gctgggtgtg gcggaccgct atcaggacat agcgttggct
4801 acccgtgata ttgctgaaga gcttggcggc gaatgggctg accgcttcct cgtgctttac
4861 ggtatcgccg ctcccgattc gcagcgcatc gccttctatc gccttcttga cgagttcttc
4921 tgagcgggac tctggggttc gaaatgaccg accaagcgac gcccaacctg ccatcacgag
4981 atttcgattc caccgccgcc ttctatgaaa ggttgggctt cggaatcgtt ttccgggacg
5041 ccggctggat gatcctccag cgcggggatc tcatgctgga gttcttcgcc caccccaact
5101 tgtttattgc agcttataat ggttacaaat aaagcaatag catcacaaat ttcacaaata
5161 aagcattttt ttcactgcat tctagttgtg gtttgtccaa actcatcaat gtatcttatc
5221 atgtctgtat accgtcgacc tctagctaga gcttggcgta atcatggtca tagctgtttc
5281 ctgtgtgaaa ttgttatccg ctcacaattc cacacaacat acgagccgga agcataaagt
5341 gtaaagcctg gggtgcctaa tgagtgagct aactcacatt aattgcgttg cgctcactgc
5401 ccgctttcca gtcgggaaac ctgtcgtgcc agctgcatta atgaatcggc caacgcgcgg
5461 ggagaggcgg tttgcgtatt gggcgctctt ccgcttcctc gctcactgac tcgctgcgct
5521 cggtcgttcg gctgcggcga gcggtatcag ctcactcaaa ggcggtaata cggttatcca
5581 cagaatcagg ggataacgca ggaaagaaca tgtgagcaaa aggccagcaa aaggccagga
5641 accgtaaaaa ggccgcgttg ctggcgtttt tccataggct ccgcccccct gacgagcatc
5701 acaaaaatcg acgctcaagt cagaggtggc gaaacccgac aggactataa agataccagg
5761 cgtttccccc tggaagctcc ctcgtgcgct ctcctgttcc gaccctgccg cttaccggat
5821 acctgtccgc ctttctccct tcgggaagcg tggcgctttc tcatagctca

```
cgctgtaggt
     5881 atctcagttc ggtgtaggtc gttcgctcca agctgggctg tgtgcacgaa
ccccccgttc
     5941 agcccgaccg ctgcgcctta tccggtaact atcgtcttga gtccaacccg
gtaagacacg
     6001 acttatcgcc actggcagca gccactggta acaggattag cagagcgagg
tatgtaggcg
     6061 gtgctacaga gttcttgaag tggtggccta actacggcta cactagaaga
acagtatttg
     6121 gtatctgcgc tctgctgaag ccagttacct tcggaaaaag agttggtagc
tcttgatccg
     6181 gcaaacaaac caccgctggt agcggttttt ttgtttgcaa gcagcagatt
acgcgcagaa
     6241 aaaaaggatc tcaagaagat cctttgatct tttctacggg gtctgacgct
cagtggaacg
     6301 aaaactcacg ttaagggatt ttggtcatga gattatcaaa aaggatcttc
acctagatcc
     6361 ttttaaatta aaaatgaagt tttaaatcaa tctaaagtat atatgagtaa
acttggtctg
     6421 acagttacca atgcttaatc agtgaggcac ctatctcagc gatctgtcta
tttcgttcat
     6481 ccatagttgc ctgactcccc gtcgtgtaga taactacgat acgggagggc
ttaccatctg
     6541 gccccagtgc tgcaatgata ccgcgagacc cacgctcacc ggctccagat
ttatcagcaa
     6601 taaaccagcc agccggaagg gccgagcgca gaagtggtcc tgcaacttta
tccgcctcca
     6661 tccagtctat taattgttgc cgggaagcta gagtaagtag ttcgccagtt
aatagtttgc
     6721 gcaacgttgt tgccattgct acaggcatcg tggtgtcacg ctcgtcgttt
ggtatggctt
     6781 cattcagctc cggttcccaa cgatcaaggc gagttacatg atcccccatg
ttgtgcaaaa
     6841 aagcggttag ctccttcggt cctccgatcg ttgtcagaag taagttggcc
gcagtgttat
     6901 cactcatggt tatggcagca ctgcataatt ctcttactgt catgccatcc
gtaagatgct
     6961 tttctgtgac tggtgagtac tcaaccaagt cattctgaga atagtgtatg
cggcgaccga
     7021 gttgctcttg cccggcgtca atacgggata ataccgcgcc acatagcaga
actttaaaag
     7081 tgctcatcat tggaaaacgt tcttcggggc gaaaactctc aaggatctta
ccgctgttga
     7141 gatccagttc gatgtaaccc actcgtgcac ccaactgatc ttcagcatct
tttactttca
     7201 ccagcgtttc tgggtgagca aaaacaggaa ggcaaaatgc cgcaaaaaag
ggaataaggg
     7261 cgacacggaa atgttgaata ctcatactct tcctttttca atattattga
agcatttatc
     7321 agggttattg tctcatgagc ggatacatat ttgaatgtat ttagaaaaat
aaacaaatag
     7381 gggttccgcg cacatttccc cgaaaagtgc cacctgacgt c
//
```
