# Supplementary material for: Identification of Critical Phosphorylation Sites Enhancing Kinase Activity With a Bimodal Fusion Framework
Source: Mol Cell Proteomics. 2024 Nov 30;24(1):100889. doi: 10.1016/j.mcpro.2024.100889 (PMC11774822; doi:10.1016/j.mcpro.2024.100889)
Supplement: Supplemental Data 8 [file mmc10.pdf]

```
LOCUS       Exported               6643 bp ds-DNA    circular SYN
22-1月-2024
DEFINITION  .
ACCESSION   .
VERSION     .
KEYWORDS    Untitled 47
SOURCE      synthetic DNA construct
  ORGANISM  synthetic DNA construct
REFERENCE   1  (bases 1 to 6643)
  AUTHORS   111111
  TITLE     Direct Submission
  JOURNAL   Exported 2024年1月22日 from SnapGene 2.3.2
            http://www.snapgene.com
FEATURES             Location/Qualifiers
     source          1..6643
                     /organism="synthetic DNA construct"
                     /mol_type="other DNA"
     enhancer        235..614
                     /note="CMV enhancer"
                     /note="human cytomegalovirus immediate early
enhancer"
     promoter        615..818
                     /note="CMV promoter"
                     /note="human cytomegalovirus (CMV) immediate
early
                     promoter"
     promoter        863..881
                     /note="T7 promoter"
                     /note="promoter for bacteriophage T7 RNA
polymerase"
     misc_feature    952..2202
                     /note="pcDNA3.1 (+) human VASP -3×MYC"
     CDS             2104..2193
                     /codon_start=1
                     /product="3 tandem Myc epitope tags"
                     /note="3xMyc"
                     /translation="EQKLISEEDLEQKLISEEDLEQKLISEEDL"
     polyA_signal    2240..2464
                     /note="bGH poly(A) signal"
                     /note="bovine growth hormone polyadenylation
signal"
     rep_origin      2510..2938
                     /direction=RIGHT
                     /note="f1 ori"
                     /note="f1 bacteriophage origin of replication;
arrow
                     indicates direction of (+) strand synthesis"
     promoter        2952..3281
                     /note="SV40 promoter"
                     /note="SV40 enhancer and early promoter"
     rep_origin      3132..3267
                     /note="SV40 ori"
                     /note="SV40 origin of replication"
     CDS             3348..4142
```

```
                     /codon_start=1
                     /gene="aph(3')-II (or nptII)"
                     /product="aminoglycoside phosphotransferase 
from Tn5"
                     /note="NeoR/KanR"
                     /note="confers resistance to neomycin, 
kanamycin, and G418 
                     (Geneticin(R))"
                     /
translation="MIEQDGLHAGSPAAWVERLFGYDWAQQTIGCSDAAVFRLSAQGRP

VLFVKTDLSGALNELQDEAARLSWLATTGVPCAAVLDVVTEAGRDWLLLGEVPGQDLLS

SHLAPAEKVSIMADAMRRLHTLDPATCPFDHQAKHRIERARTRMEAGLVDQDDLDEEHQ

GLAPAELFARLKARMPDGEDLVVTHGDACLPNIMVENGRFSGFIDCGRLGVADRYQDIA
                     LATRDIAEELGGEWADRFLVLYGIAAPDSQRIAFYRLLDEFF"
     polyA_signal    4316..4437
                     /note="SV40 poly(A) signal"
                     /note="SV40 polyadenylation signal"
     primer_bind     complement(4486..4502)
                     /note="M13 rev"
                     /note="common sequencing primer, one of 
multiple similar 
                     variants"
     protein_bind    4510..4526
                     /bound_moiety="lac repressor encoded by lacI"
                     /note="lac operator"
                     /note="The lac repressor binds to the lac 
operator to 
                     inhibit transcription in E. coli. This 
inhibition can be 
                     relieved by adding lactose or 
                     isopropyl-beta-D-thiogalactopyranoside (IPTG)."
     promoter        complement(4534..4564)
                     /note="lac promoter"
                     /note="promoter for the E. coli lac operon"
     protein_bind    4579..4600
                     /bound_moiety="E. coli catabolite activator 
protein"
                     /note="CAP binding site"
                     /note="CAP binding activates transcription in 
the presence 
                     of cAMP."
     rep_origin      complement(4888..5476)
                     /direction=LEFT
                     /note="ori"
                     /note="high-copy-number ColE1/pMB1/pBR322/pUC 
origin of 
                     replication"
     CDS             complement(5647..6507)
                     /codon_start=1
                     /gene="bla"
                     /product="beta-lactamase"
```

```
                     /note="AmpR"
                     /note="confers resistance to ampicillin, 
carbenicillin, and 
                     related antibiotics"
                     /
translation="MSIQHFRVALIPFFAAFCLPVFAHPETLVKVKDAEDQLGARVGYI

ELDLNSGKILESFRPEERFPMMSTFKVLLCGAVLSRIDAGQEQLGRRIHYSQNDLVEYS

PVTEKHLTDGMTVRELCSAAITMSDNTAANLLLTTIGGPKELTAFLHNMGDHVTRLDRW

EPELNEAIPNDERDTTMPVAMATTLRKLLTGELLTLASRQQLIDWMEADKVAGPLLRSA

LPAGWFIADKSGAGERGSRGIIAALGPDGKPSRIVVIYTTGSQATMDERNRQIAEIGAS
                     LIKHW"
     promoter        complement(6508..6612)
                     /gene="bla"
                     /note="AmpR promoter"
ORIGIN
        1 gacggatcgg gagatctccc gatcccctat ggtgcactct cagtacaatc 
tgctctgatg
       61 ccgcatagtt aagccagtat ctgctccctg cttgtgtgtt ggaggtcgct 
gagtagtgcg
      121 cgagcaaaat ttaagctaca acaaggcaag gcttgaccga caattgcatg 
aagaatctgc
      181 ttagggttag gcgttttgcg ctgcttcgcg atgtacgggc cagatatacg 
cgttgacatt
      241 gattattgac tagttattaa tagtaatcaa ttacggggtc attagttcat 
agcccatata
      301 tggagttccg cgttacataa cttacggtaa atggcccgcc tggctgaccg 
cccaacgacc
      361 cccgcccatt gacgtcaata atgacgtatg ttcccatagt aacgccaata 
gggactttcc
      421 attgacgtca atgggtggag tatttacggt aaactgccca cttggcagta 
catcaagtgt
      481 atcatatgcc aagtacgccc cctattgacg tcaatgacgg taaatggccc 
gcctggcatt
      541 atgcccagta catgacctta tgggactttc ctacttggca gtacatctac 
gtattagtca
      601 tcgctattac catggtgatg cggttttggc agtacatcaa tgggcgtgga 
tagcggtttg
      661 actcacgggg atttccaagt ctccacccca ttgacgtcaa tgggagtttg 
ttttggcacc
      721 aaaatcaacg ggactttcca aaatgtcgta acaactccgc cccattgacg 
caaatgggcg
      781 gtaggcgtgt acggtgggag gtctatataa gcagagctct ctggctaact 
agagaaccca
      841 ctgcttactg gcttatcgaa attaatacga ctcactatag ggagacccaa 
gctggctagc
      901 gtttaaactt aagcttggta ccgagctcgg atccactagt ccagtgtggt 
ggaattcgcc
      961 accatgagcg agacggtcat ctgttccagc cgggccactg tgatgcttta 
tgatgatggc
     1021 aacaagcgat ggctccctgc tggcacgggt ccccaggcct tcagccgcgt
```

ccagatctac
1081 cacaacccca cggccaattc ctttcgcgtc gtgggccgga agatgcagcc
cgaccagcag
1141 gtggtcatca actgtgccat cgtccggggt gtcaagtata accaggccac
ccccaacttc
1201 catcagtggc gcgacgctcg ccaggtctgg ggcctcaact tcggcagcaa
ggaggatgcg
1261 gcccagtttg ccgccggcat ggccagtgcc ctagaggcgt tggaaggagg
tgggccccct
1321 ccacccccag cacttcccac ctggtcggtc ccgaacggcc cctccccgga
ggaggtggag
1381 cagcagaaaa ggcagcagcc cggcccgtcg gagcacatag agcgccgggt
ctccaatgca
1441 ggaggcccac ctgctccccc cgctgggggt ccacccccac caccaggacc
tccccctcct
1501 ccaggtcccc ccccaccccc aggtttgccc ccttcggggg tcccagctgc
agcgcacgga
1561 gcagggggag gaccaccccc tgcacccccct ctcccggcag cacagggccc
tggtggtggg
1621 ggagctgggg ccccaggcct ggccgcagct attgctggag ccaaactcag
gaaagtcagc
1681 aagcaggagg aggcctcagg ggggcccaca gcccccaaag ctgagagtgg
tcgaagcgga
1741 ggtgggggac tcatggaaga gatgaacgcc atgctggccc ggagaaggaa
agccacgcaa
1801 gttggggaga aaacccccaa ggatgaatct gccaatcagg aggagccaga
ggccagagtc
1861 ccggcccaga gtgaatctgt gcggagaccc tgggagaaga acagcacaac
cttgccaagg
1921 atgaagtcgt cttcttcggt gaccacttcc gagacccaac cctgcacgcc
cagctccagt
1981 gattactcgg acctacagag ggtgaaacag gagcttctgg aagaggtgaa
gaaggaattg
2041 cagaaagtga aagaggaaat cattgaagcc ttcgtccagg agctgaggaa
gcggggttct
2101 cccgagcaga aactcatctc tgaagaagat ctggaacaaa agttgatttc
agaagaagat
2161 ctggaacaga agctcatctc tgaggaagat ctgtgactcg agtctagagg
gcccgtttaa
2221 acccgctgat cagcctcgac tgtgccttct agttgccagc catctgttgt
ttgcccctcc
2281 cccgtgcctt ccttgaccct ggaaggtgcc actcccactg tcctttccta
ataaaatgag
2341 gaaattgcat cgcattgtct gagtaggtgt cattctattc tggggggtgg
ggtggggcag
2401 gacagcaagg gggaggattg ggaagacaat agcaggcatg ctggggatgc
ggtgggctct
2461 atggcttctg aggcggaaag aaccagctgg ggctctaggg ggtatcccca
cgcgccctgt
2521 agcggcgcat taagcgcggc gggtgtggtg gttacgcgca gcgtgaccgc
tacacttgcc
2581 agcgccctag cgcccgctcc tttcgctttc ttcccttcct ttctcgccac
gttcgccggc
2641 tttccccgtc aagctctaaa tcgggggctc cctttagggt tccgatttag

tgctttacgg
2701 cacctcgacc ccaaaaaact tgattagggt gatggttcac gtagtgggcc atcgccctga
2761 tagacggttt ttcgcccttt gacgttggag tccacgttct ttaatagtgg actcttgttc
2821 caaactggaa caacactcaa ccctatctcg gtctattctt ttgatttata agggattttg
2881 ccgatttcgg cctattggtt aaaaaatgag ctgatttaac aaaaatttaa cgcgaattaa
2941 ttctgtggaa tgtgtgtcag ttagggtgtg gaaagtcccc aggctcccca gcaggcagaa
3001 gtatgcaaag catgcatctc aattagtcag caaccaggtg tggaaagtcc ccaggctccc
3061 cagcaggcag aagtatgcaa agcatgcatc tcaattagtc agcaaccata gtcccgcccc
3121 taactccgcc catcccgccc ctaactccgc ccagttccgc ccattctccg ccccatggct
3181 gactaatttt ttttatttat gcagaggccg aggccgcctc tgcctctgag ctattccaga
3241 agtagtgagg aggctttttt ggaggcctag gcttttgcaa aaagctcccg ggagcttgta
3301 tatccatttt cggatctgat caagagacag gatgaggatc gtttcgcatg attgaacaag
3361 atggattgca cgcaggttct ccggccgctt gggtggagag gctattcggc tatgactggg
3421 cacaacagac aatcggctgc tctgatgccg ccgtgttccg gctgtcagcg caggggcgcc
3481 cggttctttt tgtcaagacc gacctgtccg gtgccctgaa tgaactgcag gacgaggcag
3541 cgcggctatc gtggctggcc acgacgggcg ttccttgcgc agctgtgctc gacgttgtca
3601 ctgaagcggg aagggactgg ctgctattgg gcgaagtgcc ggggcaggat ctcctgtcat
3661 ctcaccttgc tcctgccgag aaagtatcca tcatggctga tgcaatgcgg cggctgcata
3721 cgcttgatcc ggctacctgc ccattcgacc accaagcgaa acatcgcatc gagcgagcac
3781 gtactcggat ggaagccggt cttgtcgatc aggatgatct ggacgaagag catcaggggc
3841 tcgcgccagc cgaactgttc gccaggctca aggcgcgcat gcccgacggc gaggatctcg
3901 tcgtgaccca tggcgatgcc tgcttgccga atatcatggt ggaaaatggc cgcttttctg
3961 gattcatcga ctgtggccgg ctgggtgtgg cggaccgcta tcaggacata gcgttggcta
4021 cccgtgatat tgctgaagag cttggcggcg aatgggctga ccgcttcctc gtgctttacg
4081 gtatcgccgc tcccgattcg cagcgcatcg ccttctatcg ccttcttgac gagttcttct
4141 gagcgggact ctggggttcg aaatgaccga ccaagcgacg cccaacctgc catcacgaga
4201 tttcgattcc accgccgcct tctatgaaag gttgggcttc ggaatcgttt tccgggacgc
4261 cggctggatg atcctccagc gcggggatct catgctggag ttcttcgccc

accccaactt
4321 gtttattgca gcttataatg gttacaaata aagcaatagc atcacaaatt tcacaaataa
4381 agcatttttt tcactgcatt ctagttgtgg tttgtccaaa ctcatcaatg tatcttatca
4441 tgtctgtata ccgtcgacct ctagctagag cttggcgtaa tcatggtcat agctgtttcc
4501 tgtgtgaaat tgttatccgc tcacaattcc acacaacata cgagccggaa gcataaagtg
4561 taaagcctgg ggtgcctaat gagtgagcta actcacatta attgcgttgc gctcactgcc
4621 cgctttccag tcgggaaacc tgtcgtgcca gctgcattaa tgaatcggcc aacgcgcggg
4681 gagaggcggt ttgcgtattg ggcgctcttc cgcttcctcg ctcactgact cgctgcgctc
4741 ggtcgttcgg ctgcggcgag cggtatcagc tcactcaaag gcggtaatac ggttatccac
4801 agaatcaggg gataacgcag gaaagaacat gtgagcaaaa ggccagcaaa aggccaggaa
4861 ccgtaaaaag gccgcgttgc tggcgttttt ccataggctc cgcccccctg acgagcatca
4921 caaaaatcga cgctcaagtc agaggtggcg aaacccgaca ggactataaa gataccaggc
4981 gtttccccct ggaagctccc tcgtgcgctc tcctgttccg accctgccgc ttaccggata
5041 cctgtccgcc tttctccctt cgggaagcgt ggcgctttct catagctcac gctgtaggta
5101 tctcagttcg gtgtaggtcg ttcgctccaa gctgggctgt gtgcacgaac cccccgttca
5161 gcccgaccgc tgcgccttat ccggtaacta tcgtcttgag tccaacccgg taagacacga
5221 cttatcgcca ctggcagcag ccactggtaa caggattagc agagcgaggt atgtaggcgg
5281 tgctacagag ttcttgaagt ggtggcctaa ctacggctac actagaagaa cagtatttgg
5341 tatctgcgct ctgctgaagc cagttacctt cggaaaaaga gttggtagct cttgatccgg
5401 caaacaaacc accgctggta gcggtggttt ttttgtttgc aagcagcaga ttacgcgcag
5461 aaaaaaagga tctcaagaag atcctttgat cttttctacg gggtctgacg ctcagtggaa
5521 cgaaaactca cgttaaggga ttttggtcat gagattatca aaaaggatct tcacctagat
5581 ccttttaaat taaaaatgaa gttttaaatc aatctaaagt atatatgagt aaacttggtc
5641 tgacagttac caatgcttaa tcagtgaggc acctatctca gcgatctgtc tatttcgttc
5701 atccatagtt gcctgactcc ccgtcgtgta gataactacg atacgggagg gcttaccatc
5761 tggccccagt gctgcaatga taccgcgaga cccacgctca ccggctccag atttatcagc
5821 aataaaccag ccagccggaa gggccgagcg cagaagtggt cctgcaactt tatccgcctc
5881 catccagtct attaattgtt gccgggaagc tagagtaagt agttcgccag

ttaatagttt
     5941 gcgcaacgtt gttgccattg ctacaggcat cgtggtgtca cgctcgtcgt
ttggtatggc
     6001 ttcattcagc tccggttccc aacgatcaag gcgagttaca tgatccccca
tgttgtgcaa
     6061 aaaagcggtt agctccttcg gtcctccgat cgttgtcaga agtaagttgg
ccgcagtgtt
     6121 atcactcatg gttatggcag cactgcataa ttctcttact gtcatgccat
ccgtaagatg
     6181 cttttctgtg actggtgagt actcaaccaa gtcattctga gaatagtgta
tgcggcgacc
     6241 gagttgctct tgcccggcgt caatacggga taataccgcg ccacatagca
gaactttaaa
     6301 agtgctcatc attggaaaac gttcttcggg gcgaaaactc tcaaggatct
taccgctgtt
     6361 gagatccagt tcgatgtaac ccactcgtgc acccaactga tcttcagcat
cttttacttt
     6421 caccagcgtt tctgggtgag caaaaacagg aaggcaaaat gccgcaaaaa
agggaataag
     6481 ggcgacacgg aaatgttgaa tactcatact cttccttttt caatattatt
gaagcattta
     6541 tcagggttat tgtctcatga gcggatacat atttgaatgt atttagaaaa
ataaacaaat
     6601 aggggttccg cgcacatttc cccgaaaagt gccacctgac gtc
//
